# The spatio-temporal trends and determinants of liver cancer attributable to specific etiologies: a systematic analysis from the Global Burden of Disease Study 2021

**DOI:** 10.1186/s41256-025-00416-y

**Published:** 2025-05-20

**Authors:** Jinli Liu, Tingling Xu, Yanan Wang, Fanpu Ji, Lei Zhang

**Affiliations:** 1 Department of Endocrinology, The First Affiliated Hospital of Xi’an Jiaotong University,, Xi’an Jiaotong University, Xi’an, 710061 Shaanxi People’s Republic of China; 2 China-Australia Joint Research Center for Infectious Diseases, School of Public Health, Xi’an Jiaotong University Health Science Center, Xi’an, 710061 Shaanxi People’s Republic of China; 3 Key Laboratory of Environment and Genes Related to Diseases (Xi’an Jiaotong University), Ministry of Education, Xi’an, 710061 People’s Republic of China; 4 Med-X Institute, Center for Immunological and Metabolic Diseases, the First Affiliated Hospital of Xi’an Jiaotong University, Xi’an, 710061 Shaanxi People’s Republic of China; 5 Department of Infectious Diseases, The Second Affiliated Hospital of Xi’an Jiaotong University, Xi’an, People’s Republic of China; 6 Phase I Clinical Trial Research Ward, The Second Affiliated Hospital of Xi’an Jiaotong University, No.157 Xi Wu Road, Xi’an, 710004 Shaanxi Province People’s Republic of China; 7 School of Translational Medicine, Faculty of Medicine, Nursing and Health Sciences, Monash University, Melbourne, Australia; 8 Melbourne Sexual Health Centre, Alfred Health, Melbourne, Australia

**Keywords:** Liver cancer, Prevalence, Mortality, DALYs, Trends, Decomposition analysis

## Abstract

**Background:**

Although liver cancer has varied causes, its evolving epidemiology and causal drivers remain underexplored. This study quantifies the trends and drivers of liver cancer burden attributable to specific causes from 1990 to 2021.

**Methods:**

Using data from the Global Burden of Disease Study, we extracted prevalence, mortality, and disability-adjusted life years (DALYs) associated with specific causes of liver cancer. We assessed spatio-temporal trends across the sociodemographic index (SDI) and quantified the contributions of epidemiological shifts, population growth, and ageing to DALYs.

**Results:**

In 2021, liver cancer accounted for 0.74 million cases, 0.48 million deaths, and 12.89 million DALYs globally. Average annual percentage changes (AAPCs) in prevalence, mortality, and DALY rates from 1990 to 2021 were 1.17%, 1.04%, and 0.48%, respectively. HBV, HCV, and alcohol use were major contributors to DALYs, accounting for 1.92 million (36.00%), 1.53 million (28.62%), and 1.27 million (23.88%) of the increase, respectively. High-income North America and Western Europe experienced rapid growth in liver cancer prevalence from 1990 to 2021, while High-income North America and Southern Latin America had rapid growth in mortality. Global DALY increases were mainly driven by population growth (3.91 million, 73.29%) and population ageing (3.03 million, 56.86%).

**Conclusions:**

The study revealed that hepatitis B, hepatitis C, and alcohol consumption were the primary contributors to the increasing DALYs from liver cancer, with population growth and ageing as key drivers of these changes. These findings underscore the importance of considering the major factors and demographic dynamics in addressing the burden of liver cancer when formulating prevention and intervention strategies.

**Supplementary Information:**

The online version contains supplementary material available at 10.1186/s41256-025-00416-y.

## Introduction

Liver cancer is a common malignant neoplasm worldwide. According to data from the Global Cancer Observatory, liver cancer ranks as the sixth most commonly diagnosed cancer and the third leading cause of cancer-related deaths worldwide [1]. The global disease burden of liver cancer has been increasing in recent years [2]. In regions that previously had lower liver cancer incidence, such as the United States, Australia, and parts of Europe, both incidence and mortality rates have increased. Conversely, in countries that historically had a higher burden of liver cancer, particularly in Asia and Africa, this trend has slowed and shown a declining trend [3, 4]. Between 1990 and 2019, age-standardized DALY rates for liver cancer showed a decreasing trend in East Asia and high-income Asia–Pacific regions, while they increased in Central Asia [5]. In 2020, liver cancer represented a significant global burden with 905,677 new cases and 830,180 deaths [1]. A concerning 55.0% increase in annual new cases to 1.4 million and a 56.4% rise in expected deaths to 1.3 million is projected by 2040 [6]. The rapidly increasing trend in liver cancer disease burden can be attributed to several key factors, including the high prevalence of chronic liver diseases such as hepatitis B virus (HBV) and hepatitis C virus (HCV), as well as non-alcoholic fatty liver disease [7]. Chronic HBV infection is the primary cause of HCC in East Asia and most African countries, except for Northern Africa, where HCV prevalence is highest [3, 8]. HCV is the leading virus-related cause of HCC in North America, Europe, Japan, and parts of Central Asia, including Mongolia, Northern Africa and the Middle East, particularly in Egypt [9]. Additionally, significant regional differences exist in alcohol consumption, with the World Health Organization (WHO) European Region reporting the highest levels, while the WHO Eastern Mediterranean Region has the lowest. These trends are largely influenced by cultural and religious factors [10]. Changing dietary habits, increasing alcohol consumption, and the global obesity epidemic have also contributed to the rising burden of liver cancer [11].

Efforts to mitigate the global burden of liver cancer have intensified. The WHO and the International Agency for Research on Cancer have spearheaded initiatives targeting prevention, early diagnosis, and treatment access, especially in high-burden regions, such as East Asia and North Africa [12, 13]. These initiatives include comprehensive HBV vaccination programs, enhanced surveillance and screening protocols, and public health campaigns promoting awareness of risk factors and the importance of early intervention. Socioeconomic disparities critically influence liver cancer distribution, with lower sociodemographic index (SDI) countries facing healthcare access limitations and inadequate screening and prevention programs, while higher SDI countries benefit from robust healthcare systems, enabling early diagnosis and treatment and yielding improved survival outcomes.

The global population growth, population ageing, and the distinct patterns of liver cancer development across regions have contributed to evolving epidemiological patterns and regional disparities in liver cancer burden over the past decades. To our knowledge, no comprehensive studies have quantified the spatio-temporal trends in liver cancer disease burden from global, regional, and national perspectives, nor have they examined the impact of population ageing, population growth, and the intrinsic progression of liver cancer on this burden. This study aimed to describe the spatio-temporal trends in liver cancer disease burden at the global, regional, and national levels, along with an exploration of how demographic and epidemiological factors have shaped the changes in liver cancer disease burden during 1990–2021.

## Methods

### Overview

The Global Burden of Disease Study 2021 (GBD 2021) analyzed epidemiological indicators—incidence, prevalence, mortality, and DALYs—for 371 diseases and injuries across 204 countries and territories, including subnational-level estimates for 21 countries [14]. GBD 2021 employed proportional data from a systematic literature review to assess associations between liver cancer subgroups and six factors (HBV, hepatitis C virus [HCV], alcohol use, non-alcoholic steatohepatitis [NASH], hepatoblastoma, and other causes) using several modeling approaches, details of which can be found in the appendices of the published GBD 2021 study [14]. These approaches include the Bayesian meta-regression model DisMod-MR 2.1 for synthesizing epidemiological data, meta-regression-Bayesian, regularised, trimmed (MR-BRT) for data harmonization and bias modeling, spatiotemporal Gaussian process regression (ST-GPR) for smoothing sparse data across age, time, and geography, and proportional multistate models for partitioning liver cancer burden into etiological fractions. Liver cancer cases in the GBD 2021 study were identified using International Classification of Diseases (ICD) codes C22-C22.4 and C22.7-C22.8 [14].

The 204 countries and territories were classified into five SDI regions: low, low-middle, middle, high-middle, and high. The SDI is a composite measure of lag-distributed income per capita, average years of education, and fertility rates among females under 25 years old [15]. Data on liver cancer prevalence, mortality, and DALYs were extracted for the period 1990–2021.

### Spatio-temporal analysis of disease burden of liver cancer

The Joinpoint regression model was used to analyze the temporal patterns in the disease burden associated with liver cancer, employing a grid search approach. A log-linear model was applied to assess prevalence, mortality, and DALYs of liver cancer. Trends were evaluated within independent time intervals defined by the segmentation function, using the annual percentage change (APC). The average annual percentage change (AAPC) was used to assess the global average trend across multiple time intervals.

The study applied spatial autocorrelation to examine the global spatial distribution of liver cancer burden and conducted a spatial cluster analysis for the prevalence and mortality of liver cancer in line with their AAPC. The Global Moran’s I index was used to calculate correlation statistics for all relevant spatial units within the dataset. This helped determine whether the factors exhibited spatial clustering within the studied region. Through Anselin Local Moran’s I analysis, we identified spatial clusters or regions with either elevated or reduced risk levels for the variables under investigation (see details in Supplementary material pp3).

### Decomposition analysis

The study conducted a decomposition analysis of DALYs to assess the influence of epidemiological changes in terms of population growth, population ageing, on the dynamics of liver cancer caused by specific etiologies during 1990–2021. The decomposition analysis elucidates the changes in liver cancer DALYs attributable to each factor (Detailed in the appendix methods section).

## Statistical analysis

All statistical analyses were performed using RStudio (version 4.3.3) and the Joinpoint Regression Program (version 4.9.0.0). Spatial analyses were conducted using ArcGIS version 10.2 (ESRI, Redlands, CA, USA). A p-value of < 0.05 was considered statistically significant.

## Results

### Temporal trends of liver cancer disease burden

Globally, of the 0.74 million (95% UI, 0.67–0.82) liver cancer cases in 2021, 38.97% were attributed to HBV, 27.22% to HCV, 17.86% to alcohol use, and 15.95% to 3 other factors (Table S1). The prevalence of liver cancer was 9.37 (8.53–10.42) per 100,000 population in 2021, with an AAPC of 1.17% (1.14–1.21) from 1990 to 2021 (Table 1). In 2021, the prevalence of liver cancer was highest in high SDI countries, at 21.49 (19.55–22.78) per 100,000 population, with an ASPR of 12.21 (11.3–12.86) per 100,000 population (Table 1).

**Table 1:** Global rate, and age-standardized rate of overall liver cancer prevalence in 2021 and average annual percentage change (AAPC) during 1990–2021 globally, stratified by SDI quintile and etiologies

Location	Prevalence		Age − standardized prevalence rate					
			Overall		HBV		HCV	
	Rate (/100,000, 95% UI)	AAPC (95%CI, %)	Rate (/100,000, 95% UI)	AAPC (95%CI, %)	Rate (/100,000, 95% UI)	AAPC (95%CI, %)	Rate (/100,000, 95% UI)	AAPC (95%CI, %)
Global	9.37 (8.53–10.42)	1.17 (1.14–1.21)	8.68 (7.90–9.67)	0.35 (0.31–0.39)	3.32 (2.74–4.02)	0.18 (0.14–0.23)	2.36 (2.03–2.68)	0.67 (0.59–0.76)
Low SDI	3.81 (2.80–4.98)	− 1.20 (− 1.33– − 1.07)	6.05 (4.93–7.74)	− 0.83 (− 0.91– − 0.74)	2.15 (1.60–2.84)	− 1.02 (− 1.11– − 0.94)	1.36 (1.01–1.88)	− 0.52 (− 0.59– − 0.45)
Low − middle SDI	3.90 (3.44–4.39)	0.46 (0.41–0.52)	4.74 (4.23–5.31)	0.08 (0.04–0.12)	1.35 (1.08–1.70)	− 0.17 (− 0.22– − 0.12)	1.45 (1.21–1.72)	0.29 (0.27–0.32)
Middle SDI	9.44 (8.10–11.29)	1.25 (1.15–1.35)	8.42 (7.26–9.99)	− 0.05 (− 0.15–0.05)	4.38 (3.50–5.54)	− 0.11 (− 0.21–0)	1.47 (1.21–1.78)	0.34 (0.21–0.48)
High − middle SDI	11.91 (10.16–14.14)	1.58 (1.51–1.65)	8.42 (7.21–9.96)	0.32 (0.26–0.37)	4.12 (3.27–5.18)	0.44 (0.35–0.52)	1.78 (1.51–2.10)	0.62 (0.47–0.76)
High SDI	21.49 (19.55–22.78)	2.53 (2.41–2.65)	12.21 (11.3–12.86)	1.22 (1.09–1.34)	2.86 (2.39–3.37)	0.74 (0.64–0.84)	4.54 (3.91–5.13)	1.03 (0.89–1.16)

Location	Age − standardized prevalence rate							
	Alcohol use		NASH		Hepatoblastoma		Other causes	
	Rate (/100,000, 95% UI)	AAPC (95%CI, %)	Rate (/100,000, 95% UI)	AAPC (95%CI, %)	Rate (/100,000, 95% UI)	AAPC (95%CI, %)	Rate (/100,000, 95% UI)	AAPC (95%CI, %)
Global	1.51 (1.23–1.82)	1.07 (1.00–1.13)	0.61 (0.49–0.73)	1.36 (1.32–1.41)	0.52 (0.28–0.68)	− 1.76 (− 1.94– − 1.58)	0.37 (0.3–0.44)	0.46 (0.42–0.51)
Low SDI	0.97 (0.70–1.33)	− 0.48 (− 0.61– − 0.36)	0.61 (0.43–0.83)	− 0.08 (− 0.17–0.01)	0.69 (0.17–1.02)	− 1.78 (− 1.89– − 1.66)	0.27 (0.19–0.37)	− 0.49 (− 0.56– − 0.42)
Low − middle SDI	0.79 (0.63–1.02)	0.75 (0.69–0.81)	0.48 (0.38–0.59)	1.13 (1.08–1.17)	0.48 (0.21–0.62)	− 1.42 (− 1.59– − 1.25)	0.19 (0.15–0.24)	0.35 (0.32–0.37)
Middle SDI	1.22 (0.96–1.55)	0.87 (0.82–0.91)	0.59 (0.46–0.71)	0.96 (0.84–1.07)	0.41 (0.29–0.54)	− 3.04 (− 3.47– − 2.61)	0.36 (0.28–0.45)	− 0.13 (− 0.25– − 0.02)
High − middle SDI	1.23 (0.99–1.50)	0.54 (0.49–0.6)	0.47 (0.37–0.57)	1.22 (1.17–1.28)	0.48 (0.37–0.60)	− 2.02 (− 2.66– − 1.39)	0.35 (0.27–0.44)	0.30 (0.26–0.35)
High SDI	2.82 (2.37–3.32)	1.95 (1.84–2.06)	0.82 (0.67–1.03)	2.26 (2.13–2.40)	0.59 (0.53–0.65)	0.72 (0.07–1.38)	0.57 (0.47–0.68)	1.63 (1.57–1.69)

*AAPC* average annual percentage change; *CI* confidence interval; *SDI* socio-demographic index; *UI* uncertainty interval; *HBV* hepatitis B virus; *HCV* hepatitis C virus; *NASH* non-alcohol related steatohepatitis

In 2021, global liver cancer deaths reached 0.48 million (0.44–0.54), with 37.45% attributed to HBV, 30.28% to HCV, 19.06% to alcohol use, and 13.21% to 3 other factors (Table S1). The mortality of liver cancer was 6.13 (5.58–6.85) per 100,000 population in 2021, with an AAPC of 1.04% (0.8–1.28) from 1990 to 2021 (Table 2). In 2021, high SDI countries accounted for 24.36% of global liver cancer deaths, despite representing only 13.87% of the global population (Table S1).

**Table 2:** Global rate, and age-standardized rate of overall liver cancer mortality in 2021 and average annual percentage change (AAPC) during 1990–2021 globally, stratified by SDI quintile and cause of liver cancer

Location	Mortality		Age − standardized mortality rate					
			Overall		HBV		HCV	
	Rate (/100,000, 95% UI)	AAPC (95%CI, %)	Rate (/100,000, 95% UI)	AAPC (95%CI, %)	Rate (/100,000, 95% UI)	AAPC (95%CI, %)	Rate (/100,000, 95% UI)	AAPC (95%CI, %)
Global	6.13 (5.58–6.85)	1.04 (0.80–1.28)	5.65 (5.13–6.30)	− 0.11 (− 0.35–0.12)	2.09 (1.72–2.55)	− 0.54 (− 0.81– − 0.27)	1.74 (1.49–1.99)	0.05 (− 0.26–0.36)
Low SDI	2.62 (2.14–3.33)	− 0.76 (− 0.87– − 0.64)	5.6 (4.62–7.02)	− 0.72 (− 0.81– − 0.63)	1.97 (1.47–2.62)	− 1.12( − 1.29– − 0.95)	1.62 (1.20–2.20)	− 0.52 (− 0.60– − 0.43)
Low − middle SDI	3.22 (2.93–3.57)	1.25 (1.19–1.31)	4.26 (3.88–4.7)	0.19 (0.15–0.23)	1.18 (0.95–1.49)	− 0.26 (− 0.37– − 0.14)	1.58 (1.33–1.84)	0.12 (− 0.03–0.27)
Middle SDI	6.81 (5.95–8.01)	1.27 (0.99–1.56)	6.21 (5.43–7.26)	− 0.42 (− 0.73– − 0.11)	2.92 (2.35–3.64)	− 0.89 (− 1.20– − 0.57)	1.44 (1.19–1.71)	− 0.22 (− 0.43–0)
High − middle SDI	8.26 (7.14–9.6)	1.21 (0.85–1.58)	5.53 (4.77–6.44)	− 0.28 (− 0.64–0.07)	2.50 (1.99–3.16)	− 0.38 (− 0.77–0.02)	1.48 (1.25–1.73)	− 0.19 (− 0.55–0.16)
High SDI	10.78 (9.75–11.45)	1.62 (1.41–1.83)	5.51 (5.05–5.83)	0.10 (− 0.12–0.33)	1.17 (0.97–1.40)	− 0.79 (− 1.13– − 0.46)	2.26 (1.92–2.56)	0.16 (− 0.11–0.43)

Location	Age − standardized death rate							
	Alcohol use		NASH		Hepatoblastoma		Other causes	
	Rate (/100,000, 95% UI)	AAPC (95%CI, %)	Rate (/100,000, 95% UI)	AAPC (95%CI, %)	Rate (/100,000, 95% UI)	AAPC (95%CI, %)	Rate (/100,000, 95% UI)	AAPC (95%CI, %)
Global	1.06 (0.86–1.29)	0.35 (0.22–0.48)	0.48 (0.39–0.58)	0.82 (0.72–0.91)	0.04 (0.03–0.05)	− 2.37 (− 2.56– − 2.17)	0.24 (0.19–0.29)	− 0.17 (− 0.34– − 0.01)
Low SDI	1.02 (0.74–1.40)	− 0.54 (− 0.61– − 0.46)	0.68 (0.48–0.94)	− 0.09 (− 0.17– − 0.02)	0.06 (0.04–0.08)	− 1.99 (− 2.11– − 1.88)	0.26 (0.18–0.35)	− 0.54 (− 0.63– − 0.45)
Low − middle SDI	0.80 (0.64–1.02)	0.65 (0.50–0.79)	0.50 (0.40–0.62)	1.02 (0.92–1.12)	0.04 (0.03–0.05)	− 1.71 (− 1.82– − 1.59)	0.17 (0.13–0.22)	0.24 (0.1–0.38)
Middle SDI	1.04 (0.83–1.29)	0.39 (0.26–0.52)	0.52 (0.42–0.64)	0.45 (0.21–0.69)	0.03 (0.02–0.03)	− 3.95 (− 4.26– − 3.64)	0.26 (0.20–0.32)	− 0.76 (− 1.03– − 0.49)
High − middle SDI	0.92 (0.76–1.11)	− 0.14 (− 0.54–0.27)	0.38 (0.30–0.46)	0.62 (0.35–0.90)	0.02 (0.02–0.02)	− 4.15 (− 4.54– − 3.75)	0.23 (0.18–0.28)	− 0.37 (− 0.81–0.07)
High SDI	1.36 (1.13–1.61)	0.74 (0.52–0.96)	0.44 (0.35–0.55)	1.15 (0.86–1.45)	0.02 (0.01–0.02)	− 1.10 (− 1.46– − 0.74)	0.26 (0.21–0.31)	0.48 (0.22–0.75)

*AAPC* average annual percentage change; *CI* confidence interval; *SDI* socio-demographic index; *UI* uncertainty interval; *HBV* hepatitis B virus; *HCV* hepatitis C virus; *NASH* non-alcohol related steatohepatitis

In 2021, global liver cancer DALYs reached 12.89 million (11.67–14.47), with 43.98% attributed to HBV, 24.05% to HCV, 17.97% to alcohol use, and 14.00% to 3 other factors (Table S1). The liver cancer DALYs rate was 32.28 (21.53–43.03) per 100,000 population in 2021, with an AAPC of 0.48% (0.28–0.68) from 1990 to 2021 (Table 3). In 2021, high SDI countries accounted for 31.79% of global liver cancer DALYs, despite comprising < 14% of the global population (Table S1).

**Table 3:** Global rate, and age-standardized rate of overall liver cancer DALYs in 2021 and average annual percentage change (AAPC) during 1990–2021 globally, stratified by SDI quintile and cause of liver cancer

Location	DALYs rate		Age − standardized DALYs rate					
			Overall		HBV		HCV	
	Rate (/100,000, 95% UI)	AAPC (95%CI, %)	Rate (/100,000, 95% UI)	AAPC (95%CI, %)	Rate (/100,000, 95% UI)	AAPC (95%CI, %)	Rate (/100,000, 95% UI)	AAPC (95%CI, %)
Global	32.28 (21.53–43.033)	0.48 (0.28–0.68)	149.29 (135.24–167.48)	− 0.46 (− 0.67– − 0.26)	65.36 (54.43–79.35)	− 0.79 (− 0.99– − 0.59)	35.85 (30.84–41.7)	− 0.31 (− 0.57– − 0.05)
Low SDI	86.32 (69.17–111.33)	− 0.89 (− 1.03– − 0.75)	151.07 (122.38–192.78)	− 0.8 (− 0.87– − 0.74)	60.42 (45.26–80.28)	− 1.07 (− 1.23– − 0.91)	35.69 (26.3–49.58)	− 0.59 (− 0.66– − 0.52)
Low − middle SDI	95.75 (85.95–106.47)	0.86 (0.82–0.90)	116.37 (104.91–128.97)	0.08 (0.02–0.14)	36.97 (29.77–46.5)	− 0.32 (− 0.48– − 0.16)	37.37 (31.08–44.13)	0.15 (0–0.3)
Middle SDI	193.83 (167.76–230.49)	0.73 (0.47–0.99)	169.71 (147.41–200.85)	− 0.72 (− 0.97– − 0.47)	90.66 (73.31–114.04)	− 1.01 (− 1.32– − 0.7)	30.02 (24.61–36.28)	− 0.37 (− 0.55– − 0.18)
High − middle SDI	220.67 (187.62–262.87)	0.67 (0.34–1.00)	152.6 (129.49–181.81)	− 0.63 (− 0.88– − 0.37)	80.79 (64.35–102.24)	− 0.66 (− 0.96– − 0.35)	31.09 (26.22–36.81)	− 0.44 (− 0.76– − 0.12)
High SDI	224.23 (209.27–236.5)	0.82 (0.58–1.06)	127.92 (120.57–134.41)	− 0.42 (− 0.73– − 0.12)	33.5 (28.16–39.8)	− 1.18 (− 1.54– − 0.82)	44.19 (38.25–50.45)	− 0.49 (− 0.79– − 0.2)

Location	Age − standardized DALYs rate							
	Alcohol use		NASH		Hepatoblastoma		Other causes	
	Rate (/100,000, 95% UI)	AAPC (95%CI, %)	Rate (/100,000, 95% UI)	AAPC (95%CI, %)	Rate (/100,000, 95% UI)	AAPC (95%CI, %)	Rate (/100,000, 95% UI)	AAPC (95%CI, %)
Global	26.39 (21.53–32.28)	0.18 (0.05–0.32)	11.50 (9.39–13.84)	0.60 (0.43–0.77)	3.27 (2.61–4.10)	− 2.37 (− 2.5– − 2.23)	6.92 (5.64–8.48)	− 0.47 (− 0.68– − 0.26)
Low SDI	26.02 (18.66–35.91)	− 0.56 (− 0.63– − 0.50)	16.13 (11.52–22.31)	− 0.16 (− 0.24– − 0.08)	5.27 (3.71–7.16)	− 1.99 (− 2.1– − 1.87)	7.54 (5.32–10.64)	− 0.55 (− 0.65– − 0.46)
Low − middle SDI	20.81 (16.58–26.83)	0.63 (0.43–0.82)	12.55 (10.02–15.42)	0.99 (0.89–1.10)	3.47 (2.78–4.24)	− 1.7 (− 1.81– − 1.59)	5.2 (4.07–6.61)	0.24 (0.12–0.36)
Middle SDI	26.71 (21.14–33.67)	0.26 (0.14–0.39)	12.49 (9.94–15.18)	0.24 (0.06–0.43)	2.29 (1.84–2.86)	− 3.95 (− 4.27– − 3.64)	7.54 (5.98–9.45)	− 1.05 (− 1.34– − 0.75)
High − middle SDI	23.22 (18.73–28.25)	− 0.31 (− 0.75–0.13)	9.11 (7.20–10.93)	0.30 (− 0.04–0.65)	1.66 (1.37–2.01)	− 4.13 (− 4.52– − 3.74)	6.74 (5.31–8.53)	− 0.76 (− 1.11– − 0.42)
High SDI	32.31 (27.07–38.00)	0.45 (0.23–0.67)	9.62 (7.83–11.98)	0.85 (0.53–1.16)	1.41 (1.29–1.52)	− 1.06 (− 1.42– − 0.7)	6.89 (5.74–8.28)	0.21 (0.12–0.31)

*AAPC* average annual percentage change; *CI* confidence interval; *SDI* socio-demographic index; *UI* uncertainty interval; *HBV* hepatitis B virus; *HCV* hepatitis C virus; *NASH* non-alcohol related steatohepatitis

### Liver cancer prevalence and mortality by geographical regions

Based on the results analysed in 2021, the highest prevalence and mortality of liver cancer were observed in Central Asia, East Asia, and Western Europe (Table S2). The spatial autocorrelation analysis indicated high-risk areas for liver cancer prevalence and mortality in these regions (*P* < 0.001) (Figure S1A and C). The AAPCs of liver cancer prevalence showed higher in Australasia, high-income North America, and Western Europe during 1990–2021 (Table S2), with high-risk areas identified in high-income North America and Western Europe (*P* < 0.001) (Figure S1B). The AAPCs of liver cancer mortality were higher in Australasia, High-income North America, and Southern Latin America during 1990–2021 (Table S2), with high-risk areas identified in High-income North America and Southern Latin America (*P* < 0.001) (Figure S1D).

### Drivers of liver cancer-related disability

**Fig. 1:**
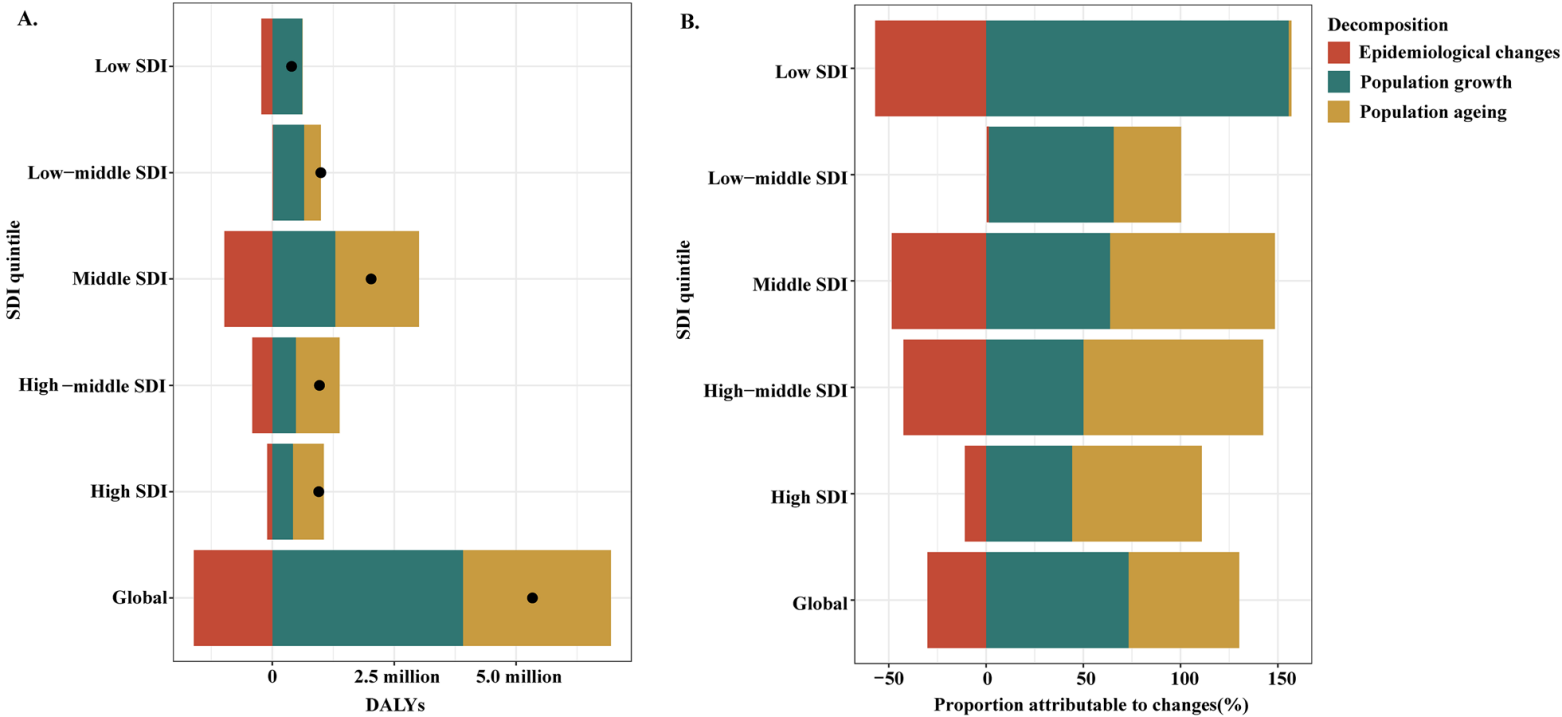
Changes in liver cancer disability adjusted life-years (DALYs) according to population-level determinants of epidemiological changes, population growth, and population ageing from 1990 to 2021 at the global level and by Sociodemographic Index (SDI) quintile (**A**). Proportion attributable to changes in liver cancer DALYs from 1990 to 2021 at the global level and by sociodemographic Index (SDI) quintile (**B**)

From the data gathered globally, population growth and population ageing contributed 3.91 million (73.29%) and 3.03 million (56.86%) to the increase in DALYs between 1990 and 2021 (Table S3, Fig. 1). The impact of population growth on liver cancer DALYs was most pronounced in the low SDI quintile, contributing 0.61 million (155.56%), while the effect of population ageing was most significant in the high-middle SDI quintile, contributing 0.89 million (92.45%) (Table S3, Fig. 1). Across ascending SDI levels, the effect of population growth declines, whereas the impact of population ageing increases.

Globally, population growth and ageing were the primary drivers of increased DALYs for liver cancer caused by specific etiologies, excluding hepatoblastoma, during 1990–2021. Population growth contributed 2.16 million (112.25%) and ageing 1.00 million (52.2%) to liver cancer due to HBV (Table S4; Figure S2A), 1.0 million (67.73%) and 0.74 million (48.18%) to HCV (Table S5; Figure S2B), 0.78 million (61.11%) and 0.42 million (32.95%) to alcohol use (Table S6; Figure S2C), 0.32 million (53.95%) and 0.16 million (27.09%) to NASH (Table S7; Figure S2D), and 0.22 million (91.91%) and 0.10 million (41.13%) to other causes (Table S9; Figure S2F), respectively. Additionally, the global burden of DALYs for liver cancer due to hepatoblastoma attributed to population growth increased by 17.00% (0.04 million), whereas the attribution by ageing decreased by 5.39% (-0.01 million) during 1990–2021 (Table S8; Figure S2E).

### Decomposition analysis by liver cancer caused by specific etiologies

From 1990 to 2021, hepatitis B virus (HBV) and hepatitis C virus (HCV) were the primary drivers of increased liver cancer DALYs globally and across all SDI levels, except in high-SDI countries. Additionally, alcohol use, NASH and other causes contributed to the increasing DALY burden of liver cancer (Table S10). Globally, liver cancer due to HBV, HCV, and alcohol use contributed 1.92 million (36.00%), 1.53 million (28.62%), and 1.27 million (23.88%) to the overall increase in DALYs during 1990–2021, respectively (Figure S3). HBV, HCV, and alcohol use were the major contributors to the increase in liver cancer DALYs across all SDI strata except high SDI countries, where HCV, alcohol use, and NASH were the more significant contributors (Table S10, Figure S3).

The study further analyzed DALYs of liver cancer caused by specific etiologies across 21 geographical regions. In East Asia, the DALYs burden of liver cancer was highest, totaling 5.06 million, with HBV accounting for 64.12% (3.25 million), HCV for 15.67% (0.79 million), and alcohol use for 9.67% (0.49 million) (Figure S4). In Oceania, the liver cancer DALY burden was the lowest at 8,992, with HBV accounting for 58.20% (5,234), HCV for 15.47% (1,391), and alcohol use for 10.58% (951) (Figure S4).

## Discussion

The study revealed that HBV, HCV, and alcohol consumption were identified as the primary risk factors contributing to the global burden of liver cancer, and were also the main drivers of elevated DALYs worldwide from 1990 to 2021. However, in high SDI countries, HCV, alcohol consumption and NASH were the leading contributors [10]. In 2021, the prevalence and mortality of liver cancer were highest in Central Asia, East Asia, and Western Europe. The high risk areas for the rapid growth of liver cancer prevalence were concentrated in high-income North America and Western Europe during 1990–2021, while Southern Latin America and high-income North America exhibited high-risk areas for the rapid growth of mortality of liver cancer. Population growth and population ageing are the primary drivers of changes in liver cancer DALYs, with population growth having the most significant impact, particularly in the low SDI quintile, where it reaches 155.56%.

The global burden of liver cancer is primarily driven by three major risk factors: hepatitis B virus (HBV), hepatitis C virus (HCV), and alcohol consumption. In 2019, HBV-related liver cancer caused 192,000 deaths [16], with disproportionate impacts in low-middle SDI regions, particularly East Asia (predominantly China) and Western Sub-Saharan Africa [2]. Despite this burden, HBV prevention remains inadequate-birth-dose vaccination coverage is still suboptimal in high-burden regions [17], while preventive and therapeutic interventions remain underutilized. HCV contributed to 300,000 annual deaths in 2020 [18], prompting WHO’s 2030 elimination targets requiring intensified screening and treatment scale-up [19]. Alcohol-related hepatocarcinogenesis was responsible for approximately 154,700 new cases (17.3%) in 2020 [20], with consumption patterns showing geographic disparity: Europe leads in per capita intake, followed by Australia and high-income North America [21]. Our analysis aligns with these patterns, demonstrating that alcohol constitutes the predominant contributor to liver cancer DALYs in high SDI countries [21]. However, beyond these well-established factors, other unhealthy lifestyle-associated risk factors may also contribute significantly role in liver cancer burden. These include environmental factors (e.g., air pollution, socio-economic stress) [22, 23], sleep disturbances (e.g., chronic sleep deprivation) [24], psychological factors (e.g., anxiety and depression) [25], physical inactivity leading to high BMI or obesity [26], exposure to harmful substances [27], and water and food contamination [28].

In 2021, the prevalence and mortality of liver cancer were highest in Central Asia, East Asia, and Western Europe. The Asian regions bearing the highest burden of liver cancer are primarily Mongolia and China. Liver cancer is the most common cancer in Mongolia, and it has the highest incidence and mortality rates [29]. The main cause of liver cancer in Mongolia is chronic infection with HBV and HCV, which together account for 98% of cases [30]. High alcohol consumption is also a contributing factor, with 47.2% of men and 27.4% of women reporting current alcohol use in 2018 [31]. The elevated disease burden of liver cancer in China can be attributed to various factors, including HBV and HCV infections [32], chronic alcohol abuse [33], environmental pollution [34], obesity [35–37], diabetes [35], and other contributing factors [32, 38, 39]. The extensive implementation of HBV vaccination has significantly contributed to reducing the burden of liver cancer in China [40]. However, the protective effect of vaccination faces challenges with delayed realization, making the immediate benefits less apparent. In developed countries in Western Europe, besides HBV and HCV, alcohol consumption is also a major factor contributing to the burden of liver cancer [20].

High-risk areas for the rapid growth of liver cancer prevalence were concentrated in High-income North America and Western Europe during 1990–2021, while high-income North America and Southern Latin America exhibited high-risk areas for the rapid growth of mortality of liver cancer. In 2015, the global prevalence of obesity among adults reached 600 million individuals with an upward trend in high-income countries [41]. The increased in diabetic cases (52.2% of type 2) has been attributed to high BMI, and projections indicate that over 1.31 billion people will have diabetes by 2050 [42, 43]. The rising prevalence of obesity is closely linked to an increase in diabetes, and both significant risk factors for metabolic dysfunction-associated steatotic liver disease and liver cancer [44–46]. However, poor healthcare conditions in South America regions have contributed to a rapid increase in liver cancer mortality, thereby adding to the disease burden of liver cancer worldwide.

The study highlighted population growth and population ageing drive changes in liver cancer DALYs, with population growth exerting the greatest impact, especially in low SDI quintiles. From 1990 to 2021, the global population has been on a steady rise, inadvertently escalating the burden of liver cancer [47]. Looking ahead, as global demographics undergo significant shifts with declining fertility rates and a growing elderly population, the burden of liver cancer attributed to population growth may gradually shift towards being influenced by aging demographics [48, 49]. The global population distribution is shifting, with population growth concentrated in less developed countries, inadvertently exacerbating the burden of liver cancer due to their expanding populations [48]. Epidemiological shifts indicate a decline in age- and population-standardized morbidity and mortality rates. However, these changes are insufficient to offset the impacts of population growth and ageing. Our findings indicate that in low-income countries, rapid population growth often correlates with increased liver cancer burden, primarily due to limited access to healthcare resources, inadequate prevention strategies, and high rates of hepatitis infections (HBV and HCV). For example, regions in sub-Saharan Africa and parts of South Asia show a significant increase in liver cancer cases alongside population growth, exacerbated by socio-economic challenges and healthcare limitations [50, 51]. Conversely, in high-income countries, while population growth is slower, aging populations contribute significantly to the burden of liver cancer. In these regions, factors such as increased alcohol consumption, lifestyle changes, and late diagnosis of liver diseases play a more prominent role. Countries like the United States and those in Western Europe have observed a rise in liver cancer cases among older populations, despite overall lower population growth rates [33]. Therefore, comprehensive strategies must be implemented to address the dual challenges of population growth and population ageing demographics particularly in high-income and low-income countries, in order to effectively reduce the global burden of liver cancer.

The study has several limitations. First, our reliance on data from GBD database implies that the limitations and uncertainties inherent in GBD estimates of liver cancer metrics are equally applicable to our study. Second, it is crucial to acknowledge potential regional and income-level disparities in the quality and availability of GBD data. This could lead to data gaps, particularly from low-income countries, and introduce biases that may affect the generalizability of our findings. Third, our assessment is at a macro-level, covering global, regional, and national epidemiologic trends of liver cancer, which may not capture micro-level trends. Fourth, although this study covers a substantial period (1990–2021), it may not fully reflect the short-term fluctuations of liver cancer trends within shorter intervals. Changes in risk factors, healthcare interventions, and public health policies over time could significantly influence the development trends of liver cancer burden. Additionally, the analysis was constrained by the incomplete availability of data on certain lifestyle-associated risk factors (e.g., air pollution, sleep patterns, mental health indicators, and food contaminants), which are acknowledged as important areas for future research.

In conclusion, the study confirms HBV, HCV, and alcohol consumption as the primary contributors to elevated liver cancer DALYs globally. Between 1990 and 2021, high-income North America and Western Europe emerged as high-risk regions for rapid increases in liver cancer prevalence, while high-income North America and Southern Latin America showed the fastest growth in liver cancer mortality. Population growth and ageing are the primary drivers of changes in liver cancer DALYs, with population growth having the greatest impact.

## Supplementary Information

Additional file1.

## Data Availability

To download the data used in these analyses, please visit the Global Health Data Exchange at https://vizhub.healthdata.org/gbd-results/.

## Abbreviations

AAPC: Average annual percentage change
ASMR: Age-standardized mortality rate
ASPR: Age-standardized prevalence rate
DALYs: Disability-adjusted life-years
GBD: Global burden of disease
HBV: Hepatitis B virus
HCV: Hepatitis C virus
NASH: Non-alcohol related steatohepatitis
SDI: Socio-demographic index
UI: Uncertainty interval
