# Supplementary material for: The spatio-temporal trends and determinants of liver cancer attributable to specific etiologies: a systematic analysis from the Global Burden of Disease Study 2021
Source: Glob Health Res Policy. 2025 May 20;10:22. doi: 10.1186/s41256-025-00416-y (PMC12090427; doi:10.1186/s41256-025-00416-y)
Supplement: Supplementary file 1 — Additional file1. [file 41256_2025_416_MOESM1_ESM.pdf]

Supplemental Materials

The spatio-temporal trends and determinants of liver cancer attributable to specific etiologies: a systematic analysis from the Global Burden of Disease Study 2021

This appendix provided more methodological detail on spatial autocorrelation analysis, decomposition analysis and relative results.

Contents

1. Supplementary Methods……………………………………………………………………………………3

1.1 Spatial autocorrelation analysis………………………………………………………………………………3

1.2 Decomposition analysis…………………………………………………………………………………………4

References……4

2. Supplementary Figures……………………………………………………………………………………5

Figure S1. Country-level variation and local Moran-spatial autocorrelation analysis of prevalence, mortality and their average annual percentage change (AAPCs) of liver cancer during 1990-2021……………………………………………………5

Figure S2. Changes in liver cancer caused by specific etiologies disability adjusted life-years (DALYs) according to population-level determinants of epidemiological change, population growth, and aging from 1990 to 2021 at the global level and by sociodemographic index (SDI) quintile ………6

Figure S3. The changes (A) and proportion attributable to changes from 1990 to 2021 (B) of disability adjusted life-years (DALYs) of liver cancer according to the 6 causes at the global level and by sociodemographic Index (SDI) quintile.……………………………10

Figure S4. The number of disability adjusted life-years (DALYs) of liver cancer caused by specific etiologies from 1990 to 2021 by geographical regions……………………………………………………………………………………11

3. Supplementary Tables……………………………………………………………………………………12

Table S1. The number of prevalence, mortality and disability adjusted life-years (DALYs) of liver cancer and due to other factors during 1990–2021 at the global region……………………………………………………………………………12

Table S2. The disease burden of prevalence, mortality and disability adjusted life-years (DALYs) of liver cancer and their average annual percentage

change (AAPCs) during 1990–2021 at the global region……15
Table S3. Changes in disability adjusted life-years (DALYs) number of liver cancer according to population-level determinants of aging, population growth, and epidemiological change from 1990 to 2021 at the global level and by sociodemographic index (SDI) quintile……19
Table S4. Changes in disability adjusted life-years (DALYs) number of liver cancer due to hepatitis B according to population-level determinants of aging, population growth, and epidemiological change from 1990 to 2021 at the global level and by sociodemographic index (SDI) quintile……20
Table S5. Changes in disability adjusted life-years (DALYs) number of liver cancer due to hepatitis C according to population-level determinants of aging, population growth, and epidemiological change from 1990 to 2021 at the global level and by sociodemographic index (SDI) quintile……21
Table S6. Changes in disability adjusted life-years (DALYs) number of liver cancer due to alcohol use according to population-level determinants of aging, population growth, and epidemiological change from 1990 to 2021 at the global level and by sociodemographic index (SDI) quintile……22
Table S7. Changes in disability adjusted life-years (DALYs) number of liver cancer due to NASH according to population-level determinants of aging, population growth, and epidemiological change from 1990 to 2021 at the global level and by sociodemographic index (SDI) quintile……23
Table S8. Changes in disability adjusted life-years (DALYs) number of liver cancer due to hepatoblastoma according to population-level determinants of aging, population growth, and epidemiological change from 1990 to 2021 at the global level and by sociodemographic index (SDI) quintile……24
Table S9. Changes in disability adjusted life-years (DALYs) number of liver cancer due to other causes according to population-level determinants of aging, population growth, and epidemiological change from 1990 to 2021 at the global level and by sociodemographic index (SDI) quintile……25
Table S10. Changes in disability adjusted life-years (DALYs) number of liver cancer caused by specific etiologies from 1990 to 2021 at the global level and by sociodemographic index (SDI) quintile……26

1. Supplementary Methods

1.1 Spatial autocorrelation analysis

We applied spatial autocorrelation to examine the global spatial distribution of liver cancer prevalence, mortality, and their AAPC with the global spatial autocorrelation index Moran's I in Arcgis 10.2[1]. Spatial cluster analysis utilized the Global Moran's I index to determine spatial clustering within the dataset. Anselin Local Moran's I analysis identified regions with elevated or reduced risk levels. Moran's index was defined as follows:

$$I=\frac{n\sum_{i=1}^{n}\sum_{j=1}^{n}w_{ij}\left(x_i-\bar{x}\right)\left(x_j-\bar{x}\right)}{\sum_{i=1}^{n}\sum_{j=1}^{n}w_{ij}\sum_{i=0}^{n}\left(x_i-\bar{x}\right)^2} \qquad (1)$$

where n is the number of spatial units, x_i and x_j are the value of the variable in the region i, x is the arithmetic average for the given variables, and w_{ij} is the spatial weight. Moran's index (I) varies between -1 and +1. I>0 indicates a positive autocorrelation, the closer to 1, the stronger the spatial aggregation of the prevalence and mortality of liver cancer, as well as their AAPC. I<0 indicates a negative spatial autocorrelation, the closer to -1, the greater the spatial heterogeneity of those indicators. I=0 means that liver cancer are randomly distributed and do not have spatial autocorrelation.

A local index of spatial autocorrelation (LISA, represented by a local Moran's index) identifies the presence of spatial clusters with high or low values. It identifies the specific location of the aggregation[2]. Five categorizations are possible for LISA: (1) high-high; (2) low-low; (3) high-low; (4) low-high; and (5) no significant local spatial autocorrelation. The local Moran's index is defined as:

$$I=\frac{n^2\left(x_i-\bar{x}\right)\sum_{i=1}^{n}\sum_{j=1}^{n}w_{ij}\left(x_j-\bar{x}\right)}{\sum_{i=1}^{n}\sum_{j=1}^{n}w_{ij}\sum_{i=0}^{n}\left(x_j-\bar{x}\right)^2} \qquad (2)$$

1.2 Decomposition analysis

We applied the decomposition methodology introduced by Das Gupta[3] to disentangle liver cancer and 6 causes of liver cancer DALYs by considering epidemiological changes, population growth, population ageing. The calculation of DALYs for each specific location was accomplished employing the subsequent formula:

$$DALY_{ay,\,py,\,ey}=\sum_{i=1}^{20}(a_{i,y} * p_y * e_{i,y})$$

Herein, $DALY_{ay,\,py,\,ey}$ delineates the DALYs attributed to the combined factors of age structure,

population, and DALYs rate for a specific year denoted as y; where $a_{i,y}$ signifies the proportion of the population falling within age category i of the 20 age categories in the given year y; p_y denotes the total population for the specific year y; and $e_{i,y}$ signifies the DALYs rate pertaining to age category i during the year y. The quantified impact of each individual factor on the change in DALYs between the years 1990 and 2021 was discerned through the variation of one factor, while keeping all other variables constant. For instance, the influence of age structure was calculated as follows:

$[(DALY_{a2021, p1990, e1990} + DALY_{a2021, p2021, e2021})/3 + (DALY_{a2021, p1990, e2021} + DALY_{a2021, p2021, e1990})/6] - [(DALY_{a1990, p2021, e2021} + DALY_{a1990, p1990, e1990})/3 + (DALY_{a1990, p2021b, e1990} + DALY_{a1990, p1990, e2021})/6]$

References:

1.Odland, J. Spatial Autocorrelation; Department of Geography: Bloomington, IN, USA, 1988.
2.Anselin, L. Local indicators of spatial association-LISA. Geogr. Anal. 1995, 27, 93–115.
3. Chevan A, Sutherland M. Revisiting Das Gupta: refinement and extension of standardization and decomposition. Demography 2009; 46(3): 429-49.

2. Supplementary Figures

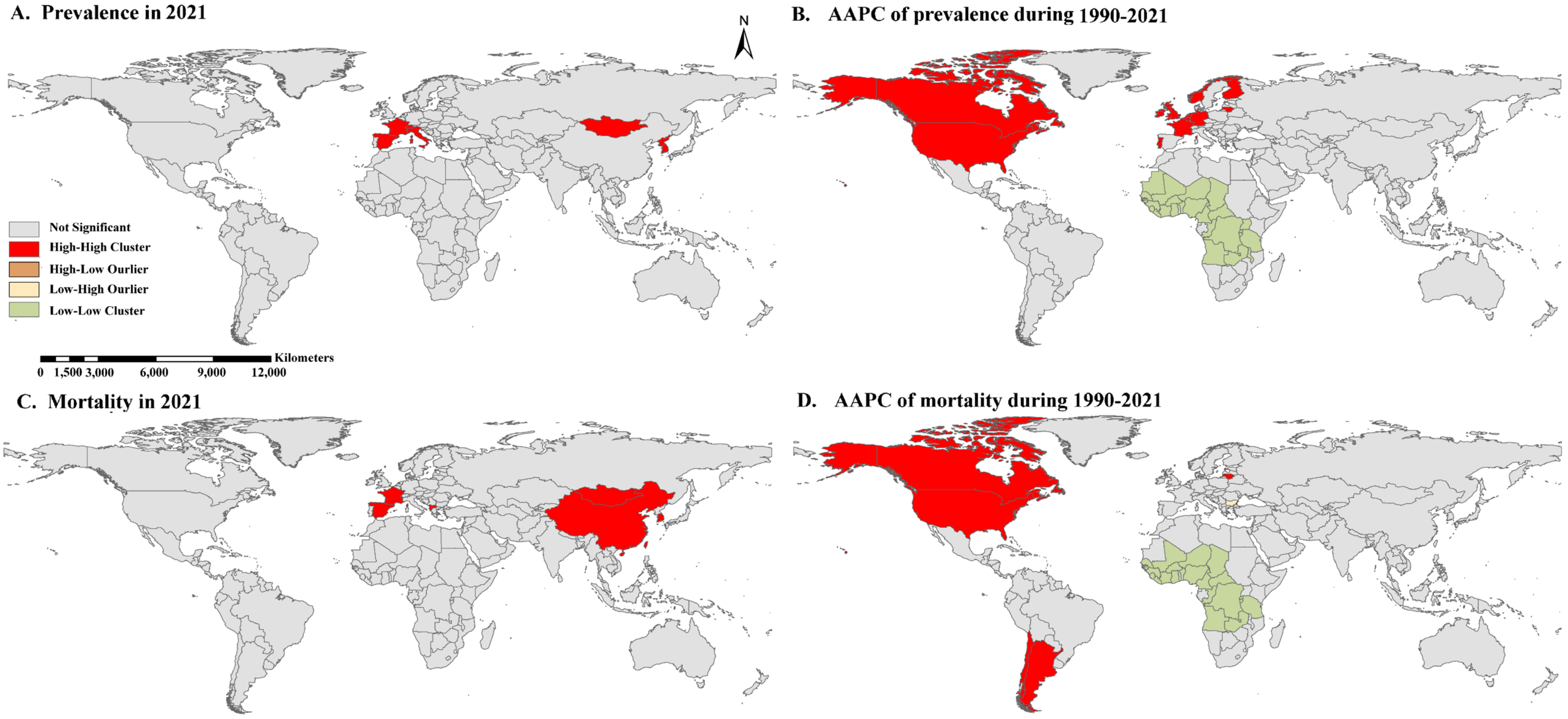

Figure S1. Country-level variation and local Moran-spatial autocorrelation analysis of prevalence, mortality and their average annual percentage change (AAPCs) of liver cancer during 1990-2021

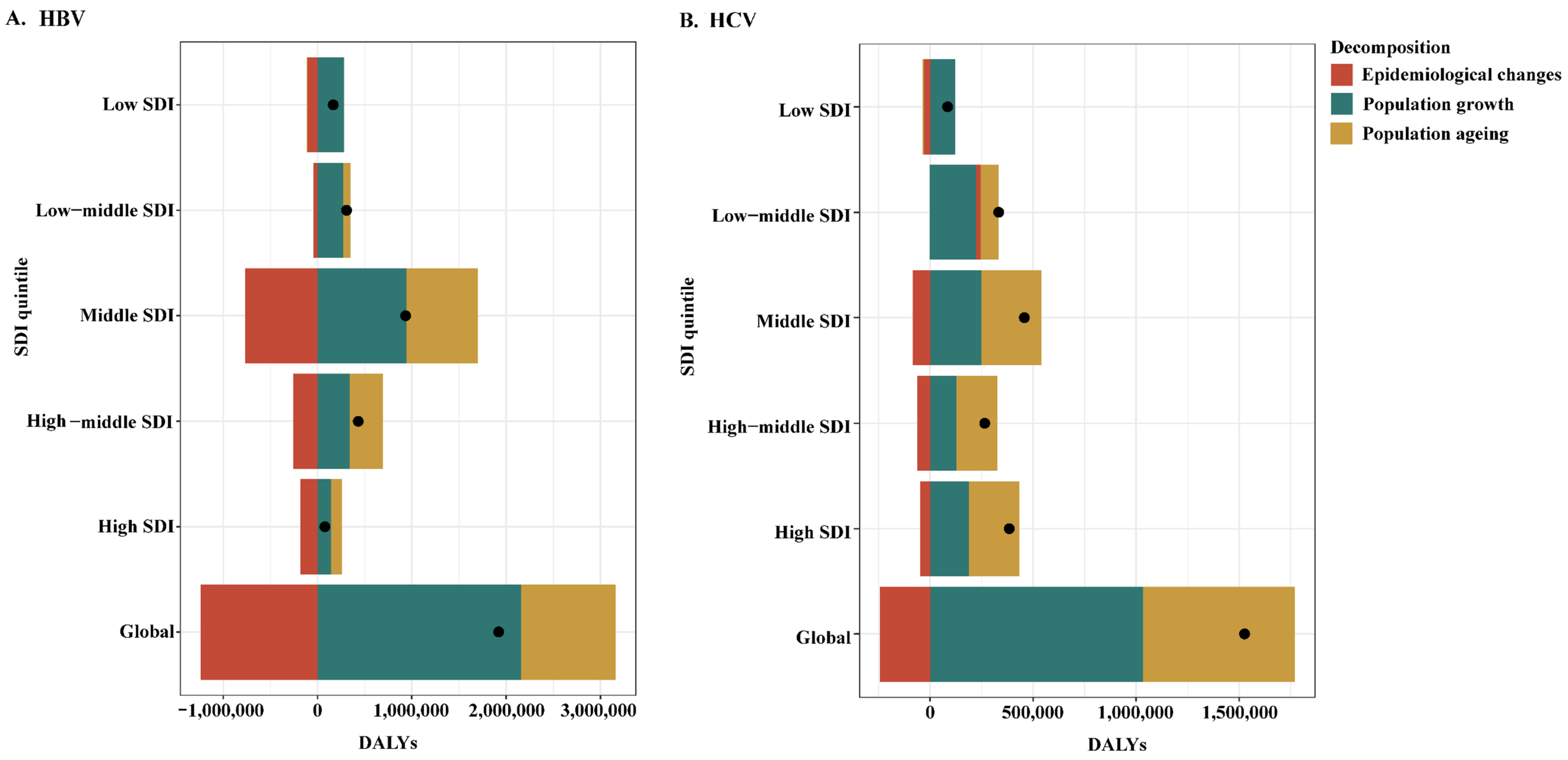
A. HBV
B. HCV
SDI quintile
Low SDI
Low−middle SDI
Middle SDI
High−middle SDI
High SDI
Global
−1,000,000
0
1,000,000
2,000,000
3,000,000
500,000
1,500,000
DALYs
Decomposition
Epidemiological changes
Population growth
Population ageing

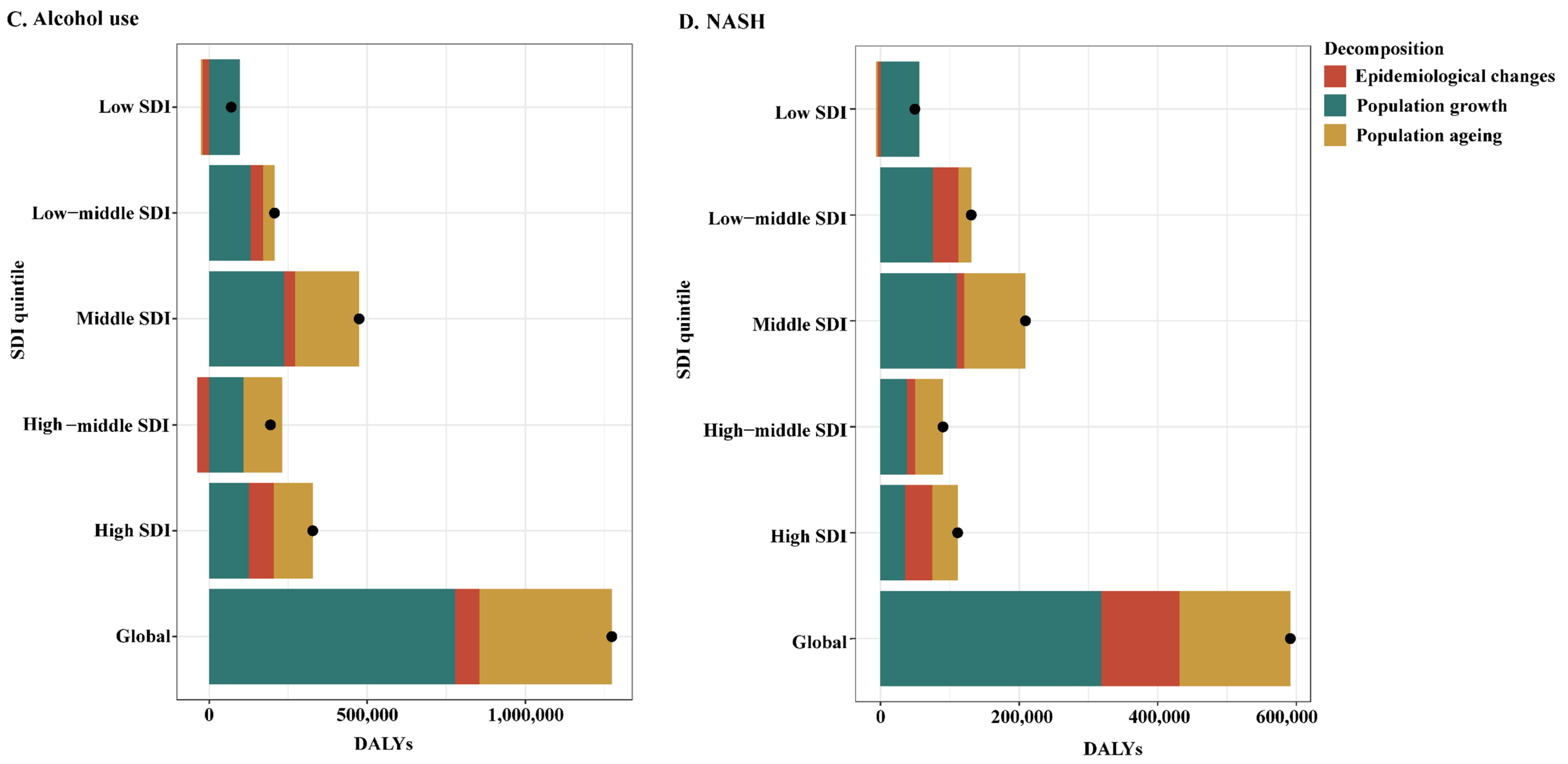
C. Alcohol use
SDI quintile
Low SDI
Low−middle SDI
Middle SDI
High−middle SDI
High SDI
Global
0
500,000
1,000,000
DALYs
D. NASH
SDI quintile
Low SDI
Low−middle SDI
Middle SDI
High−middle SDI
High SDI
Global
0
200,000
400,000
600,000
DALYs
Decomposition
Epidemiological changes
Population growth
Population ageing

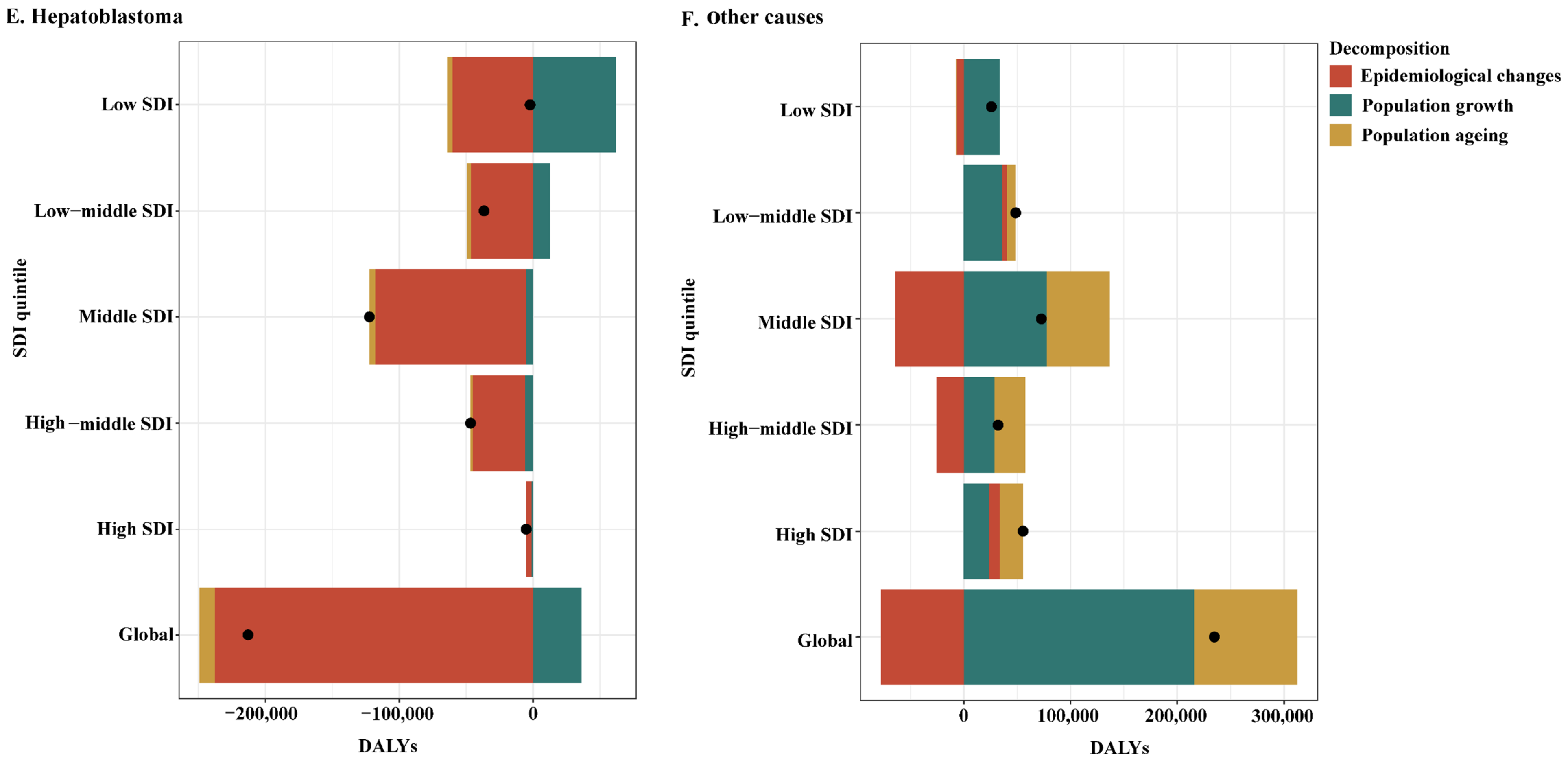

Figure S2. Changes in liver cancer caused by specific etiologies (A. Hepatitis B [HBV], B. Hepatitis C [HCV], C. Alcohol use, D. NASH, E. Hepatoblastoma, F. Other causes) disability adjusted life-years (DALYs) according to population-level determinants of epidemiological changes, population growth, and population ageing from 1990 to 2021 at the global level and by sociodemographic Index (SDI) quintile (The black dot represents the overall value of change contributed by all 3 components. For each component, the magnitude of a positive value indicates a corresponding increase in liver cancer DALYs attributed to the component; the magnitude of a negative value indicates a corresponding decrease in liver cancer DALYs attributed to the related component).

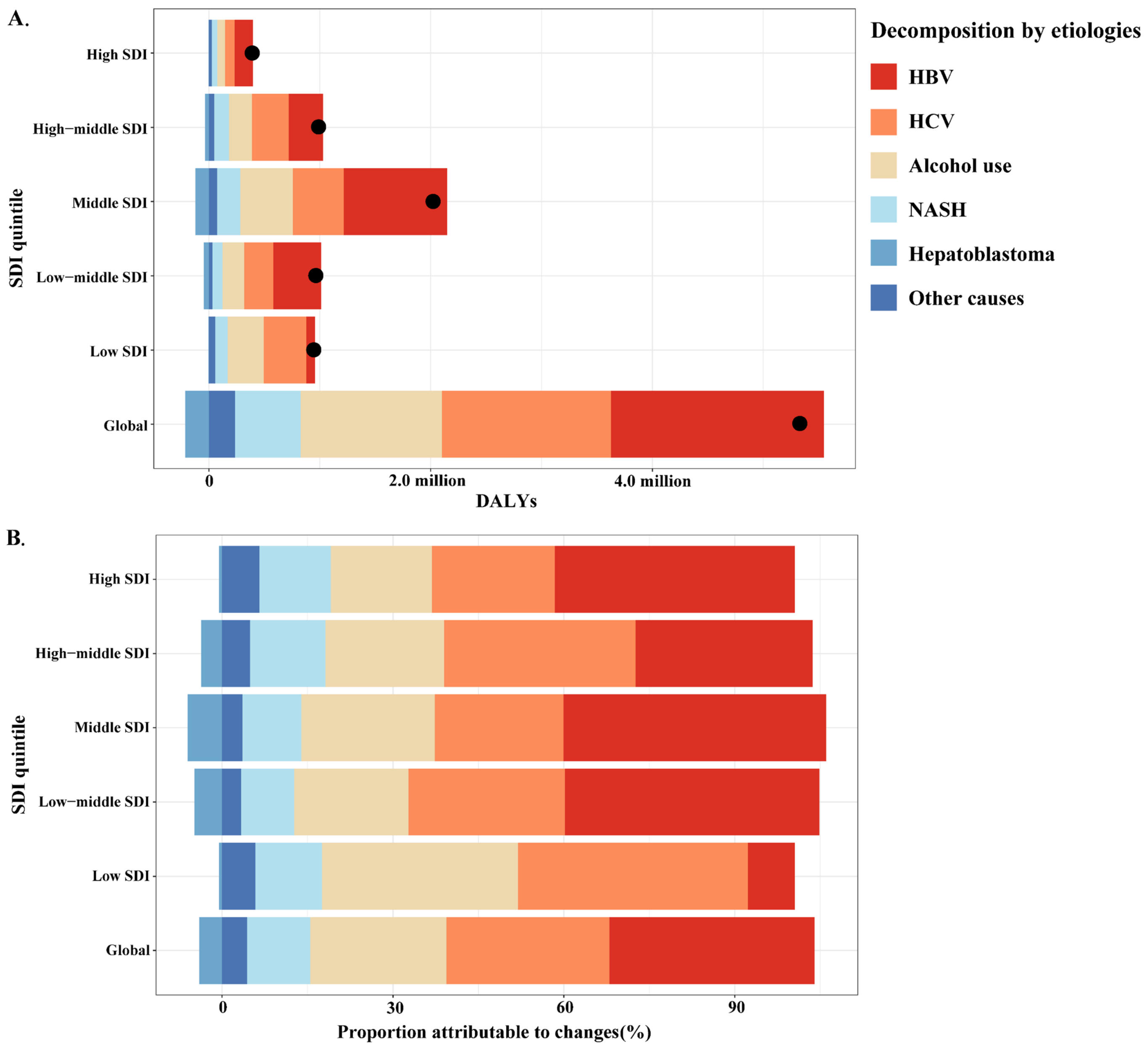

Figure S3. The changes (A) and proportion attributable to changes from 1990 to 2021 (B) of disability adjusted life-years (DALYs) of liver cancer according to the 6 causes at the global level and by sociodemographic Index (SDI) quintile. The black dot represents the overall value of change contributed by all 6 etiologies. For each component, the magnitude of a positive value indicates a corresponding increase in liver cancer DALYs attributed to the component; the magnitude of a negative value indicates a corresponding decrease in liver cancer DALYs attributed to the related component (HBV: Hepatitis B virus; HCV: Hepatitis C virus; NASH: Non-alcohol related steatohepatitis).

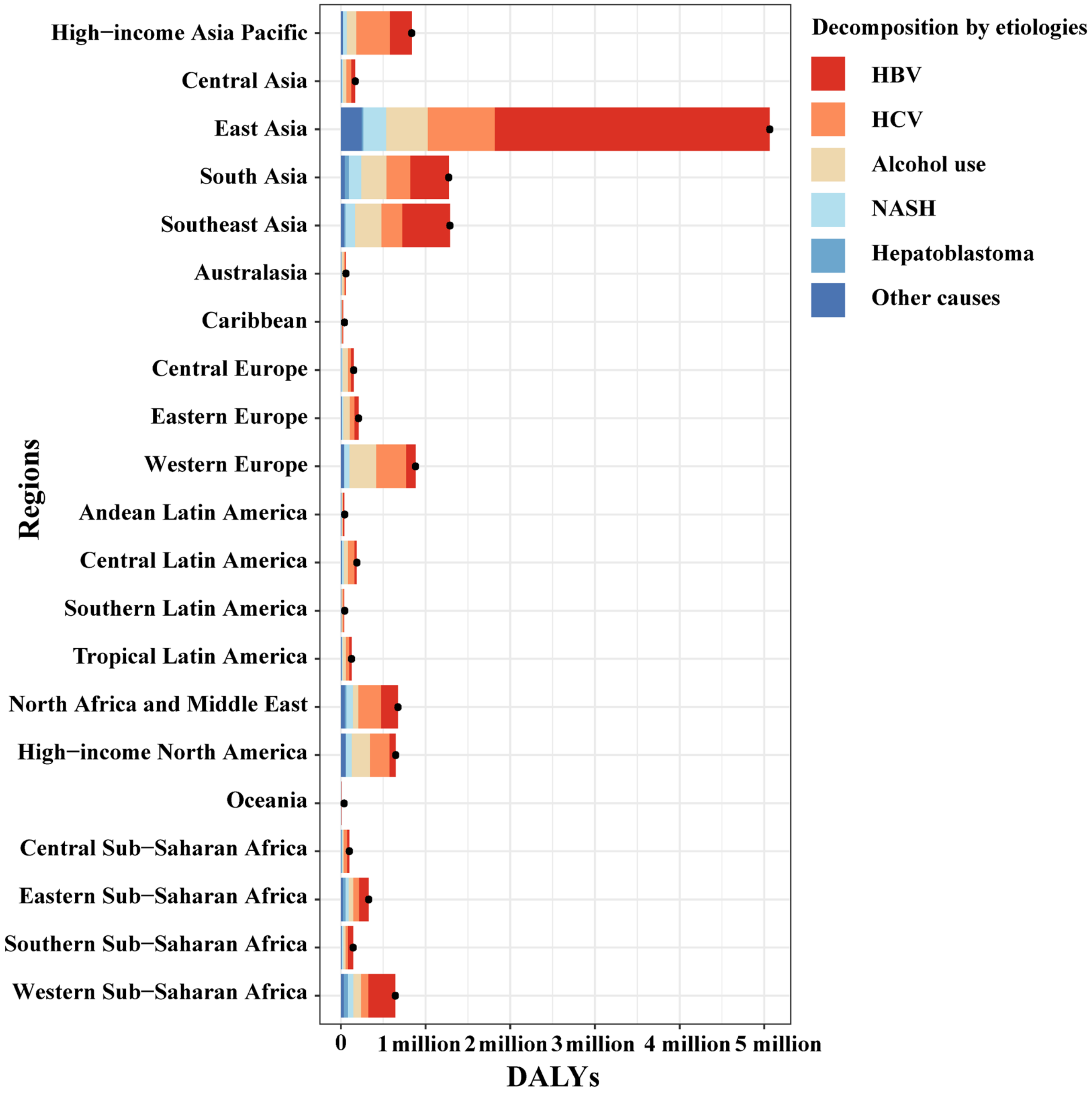

Figure S4. The number of disability adjusted life-years (DALYs) of liver cancer caused by specific etiologies from 1990 to 2021 by geographical regions. (HBV: Hepatitis B virus; HCV: Hepatitis C virus; NASH: Non-alcohol related steatohepatitis)

3. Supplementary Tables

Table S1. The number of prevalence cases, deaths and disability adjusted life-years (DALYs) of liver cancer and due to other factors during 1990–2021 at the global region and by socio-demographic index (SDI) quintile

	Prevalence cases (No. ×10^3, 95%UI)			Deaths (No. ×10^3, 95%UI)			DALYs number (No. ×10^3, 95%UI)	
	1990	2021		1990	2021		1990	2021
**Global**								
Overall	345.91(299.83–376.63)	739.3(673.11–821.95)		238.97(218.72–263.04)	483.88(440.4–540.18)		7553.67(6897.51–8296.18)	12887.65(11673.53–14472.23)
HBV	138.79(121.37–160.87)	288.11(237.81–349.75)		106.51(91.94–124.29)	181.19(148.9–221.69)		3748.18(3255.63–4362.51)	5668.2(4706.89–6885.07)
HBV	75.73(66.44–87.31)	201.21(173.23–228.76)		64.13(55.3–75.52)	146.52(125.94–168.52)		1572.21(1347.33–1870.51)	3098.87(2662.3–3609.08)
Alcohol use	44.85(37.05–54.44)	132.03(107.26–159.05)		38.17(31.17–46.2)	92.23(75.05–112.16)		1042.12(852.87–1280.54)	2316.03(1887.01–2845.79)
NASH	16.45(13.19–20.26)	52.43(42.49–63.2)		14.68(11.62–18.16)	40.92(32.96–49.61)		404.01(321.35–499.99)	995.47(808.8–1201.79)
Hepatoblastoma	56.18(20.48–73.95)	33.83(18.01–44.47)		4.83(3.94–5.67)	2.42(1.92–3.02)		426.36(348.86–500.16)	213.48(170.09–267.25)
Other causes	13.91(11.56–17.07)	31.69(25.74–38.47)		10.65(8.7–13.33)	20.59(16.37–25.05)		360.79(299.26–444.15)	595.6(484.8–732.31)
**Low SDI Countries**								
Overall	27.8(17.09–34.91)	42.6(31.33–55.64)		16.56(12.29–21.84)	29.33(23.88–37.22)		27.8(17.09–34.91)	42.6(31.33–55.64)
HBV	8.09(5.35–11.43)	13.95(10.53–18.46)		6.89(4.54–9.89)	11.35(8.48–14.99)		8.09(5.35–11.43)	13.95(10.53–18.46)
HBV	3.56(2.43–5.34)	6.81(4.96–9.48)		3.78(2.6–5.7)	7.2(5.35–9.94)		3.56(2.43–5.34)	6.81(4.96–9.48)
Alcohol use	2.71(1.75–3.94)	5.31(3.8–7.26)		2.66(1.73–3.89)	5.12(3.7–7.02)		2.71(1.75–3.94)	5.31(3.8–7.26)

NASH	1.48(0.97–2.22)	3.3(2.38–4.46)		1.46(0.94–2.24)	3.22(2.28–4.45)		1.48(0.97–2.22)	3.3(2.38–4.46)
Hepatoblastoma	11.09(2.44–16.51)	11.49(2.92–16.9)		1.01(0.7–1.31)	0.99(0.7–1.34)		11.09(2.44–16.51)	11.49(2.92–16.9)
Other causes	0.87(0.58–1.34)	1.74(1.26–2.45)		0.74(0.49–1.16)	1.45(1.03–2.02)		0.87(0.58–1.34)	1.74(1.26–2.45)
**Low-middle SDI Countries**								
Overall	39.21(27.88–45.65)	74.89(66.17–84.28)		25.49(21.93–31.21)	61.94(56.19–68.55)		39.21(27.88–45.65)	74.89(66.17–84.28)
HBV	10.57(8.26–13.57)	22.17(17.78–27.8)		8.69(6.81–11.28)	18.25(14.6–23.06)		10.57(8.26–13.57)	22.17(17.78–27.8)
HBV	8.21(6.29–11.24)	21.32(17.67–25.36)		8.47(6.48–11.61)	21.73(18.08–25.54)		8.21(6.29–11.24)	21.32(17.67–25.36)
Alcohol use	4.11(3.14–5.45)	12.04(9.61–15.44)		3.96(3.05–5.16)	11.58(9.28–14.97)		4.11(3.14–5.45)	12.04(9.61–15.44)
NASH	2.23(1.69–3.06)	7.24(5.8–8.91)		2.14(1.57–3.01)	7.04(5.61–8.68)		2.23(1.69–3.06)	7.24(5.8–8.91)
Hepatoblastoma	12.82(3.16–18.82)	9.04(3.98–11.92)		1.17(0.79–1.47)	0.75(0.6–0.92)		12.82(3.16–18.82)	9.04(3.98–11.92)
Other causes	1.27(0.94–1.8)	3.07(2.4–3.93)		1.06(0.78–1.53)	2.59(2–3.3)		1.27(0.94–1.8)	3.07(2.4–3.93)
**Middle SDI Countries**								
Overall	113.38(96.52–128.05)	231.13(198.44–276.54)		79.63(69.97–89.5)	166.73(145.74–196.16)		113.38(96.52–128.05)	231.13(198.44–276.54)
HBV	58.49(49.84–68.57)	124.47(99.28–157.94)		45.53(38.95–52.95)	81.09(64.9–101.64)		58.49(49.84–68.57)	124.47(99.28–157.94)
HBV	13.52(11.24–16.18)	39.33(32.1–47.7)		14.13(11.79–16.74)	36.23(29.91–43.32)		13.52(11.24–16.18)	39.33(32.1–47.7)
Alcohol use	10.47(8.43–13.19)	34.29(26.8–43.89)		9.71(7.87–12.15)	28.33(22.39–35.63)		10.47(8.43–13.19)	34.29(26.8–43.89)
NASH	4.95(4.04–5.99)	15.98(12.55–19.51)		4.59(3.71–5.6)	13.61(10.82–16.68)		4.95(4.04–5.99)	15.98(12.55–19.51)
Hepatoblastoma	21.13(7.49–27.05)	7(5.04–9.32)		1.84(1.6–2.13)	0.46(0.37–0.57)		21.13(7.49–27.05)	7(5.04–9.32)
Other causes	4.82(3.97–5.93)	10.05(7.86–12.74)		3.83(3.12–4.71)	7.01(5.48–8.75)		4.82(3.97–5.93)	10.05(7.86–12.74)
**High-middle SDI Countries**								
Overall	78.56(68.89–88.62)	155.3(132.5–184.37)		60.54(53.67–67.8)	107.71(93.07–		78.56(68.89–88.62)	155.3(132.5–184.37)

					125.15)			
HBV	38.33(32.06–45.24)	76.54(60.53–96.59)		29.65(24.7–35.26)	47.9(37.91–60.44)		38.33(32.06–45.24)	76.54(60.53–96.59)
HBV	14.61(12.49–17.05)	35.45(29.91–41.86)		14.67(12.56–17.07)	29.37(24.76–34.38)		14.61(12.49–17.05)	35.45(29.91–41.86)
Alcohol use	10.73(8.93–12.9)	24.46(19.55–29.82)		9.8(8.13–11.66)	18.54(15.11–22.32)		10.73(8.93–12.9)	24.46(19.55–29.82)
NASH	3.33(2.73–4.01)	9.21(7.29–11.16)		3.1(2.54–3.75)	7.44(5.95–9.09)		3.33(2.73–4.01)	9.21(7.29–11.16)
Hepatoblastoma	8.23(4.83–10.31)	3.17(2.46–4.03)		0.66(0.57–0.77)	0.13(0.11–0.16)		8.23(4.83–10.31)	3.17(2.46–4.03)
Other causes	3.34(2.7–4.13)	6.46(5.06–8.19)		2.65(2.13–3.29)	4.34(3.38–5.39)		3.34(2.7–4.13)	6.46(5.06–8.19)
**High SDI Countries**								
Overall	86.78(82.35–90.71)	235.06(213.88–249.19)		56.59(53.36–59.79)	117.89(106.69–125.24)		86.78(82.35–90.71)	235.06(213.88–249.19)
HBV	23.25(19.79–27.23)	50.88(41.69–60.88)		15.7(13.14–18.51)	22.54(18.39–27.2)		23.25(19.79–27.23)	50.88(41.69–60.88)
HBV	35.79(32.63–39.16)	98.21(83.59–111.14)		23.04(20.76–25.6)	51.92(43.97–58.95)		35.79(32.63–39.16)	98.21(83.59–111.14)
Alcohol use	16.79(14.26–19.51)	55.84(46.49–65.54)		11.99(10.07–14.06)	28.57(23.77–33.86)		16.79(14.26–19.51)	55.84(46.49–65.54)
NASH	4.45(3.62–5.48)	16.67(13.37–20.74)		3.36(2.66–4.2)	9.58(7.41–11.97)		4.45(3.62–5.48)	16.67(13.37–20.74)
Hepatoblastoma	2.9(2.55–3.16)	3.11(2.8–3.43)		0.14(0.13–0.16)	0.09(0.08–0.09)		2.9(2.55–3.16)	3.11(2.8–3.43)
Other causes	3.6(3.02–4.29)	10.34(8.4–12.48)		2.36(1.93–2.87)	5.19(4.14–6.32)		3.6(3.02–4.29)	10.34(8.4–12.48)

Note: DALYs: Disability-adjusted life-years; SDI: Socio-demographic index; UI: Uncertainty interval; HBV: Hepatitis B virus; HCV: Hepatitis C virus; NASH: Non-alcohol related steatohepatitis.

Table S2. The disease burden of prevalence, mortality and disability adjusted life-years (DALYs) of liver cancer and their average annual percentage changes (AAPCs) during 1990–2021 by geographical regions

Characteristics	1990			2021			1990-2021
	Cases	Rate per 100 000		Cases	Rate per 100 000		AAPC
	No.×10^3 (95% UI)	No.(95% UI)		No.×10^3 (95% UI)	No.(95% UI)		No.(95% CI)
**Prevalence**							
High-income Asia Pacific	53.97(49.69–57.93)	31.13(28.66–33.41)		108.8(94.72–119.76)	58.67(51.07–64.58)		2.05(1.93–2.18)
Central Asia	4.83(4.18–5.47)	6.97(6.03–7.89)		6.48(5.5–7.59)	6.77(5.74–7.92)		-0.13(-0.25–0)
East Asia	136.98(112.97–159.08)	11.25(9.28–13.07)		275.11(222.24–340.13)	18.68(15.09–23.09)		1.72(1.6–1.85)
South Asia	22.97(15.57–28.24)	2.1(1.42–2.58)		52.28(45.78–58.69)	2.83(2.48–3.18)		0.98(0.9–1.05)
Southeast Asia	26.45(22.41–30.32)	5.68(4.81–6.51)		53.64(42.3–69.36)	7.68(6.06–9.93)		0.98(0.94–1.01)
Australasia	0.6(0.55–0.64)	2.94(2.72–3.17)		4.5(4.03–5.01)	14.53(13.01–16.19)		5.3(5.13–5.47)
Caribbean	0.63(0.55–0.72)	1.79(1.55–2.03)		1.1(0.95–1.26)	2.31(1.99–2.66)		0.82(0.74–0.89)
Central Europe	4.94(4.55–5.35)	3.95(3.64–4.28)		6.59(5.92–7.34)	5.72(5.13–6.37)		1.21(1.13–1.3)
Eastern Europe	6.38(6.07–6.69)	2.82(2.68–2.96)		8.84(8.2–9.56)	4.28(3.96–4.62)		1.22(0.88–1.57)
Western Europe	24.26(23.27–25.21)	6.31(6.05–6.56)		71.75(66.42–76.18)	16.41(15.19–17.42)		3.12(3.06–3.18)
Andean Latin America	0.89(0.64–1.08)	2.34(1.69–2.85)		1.69(1.31–2.13)	2.55(1.98–3.22)		0.3(0.14–0.45)
Central Latin America	3.85(2.92–4.23)	2.34(1.78–2.57)		7.99(7.13–8.91)	3.16(2.82–3.52)		0.98(0.92–1.05)
Southern Latin America	0.41(0.37–0.47)	0.84(0.74–0.95)		1.68(1.51–1.85)	2.48(2.24–2.74)		3.57(3.49–3.64)
Tropical Latin America	2.26(1.84–2.49)	1.48(1.21–1.63)		5.23(4.92–5.51)	2.3(2.16–2.42)		1.46(1.35–1.57)
North Africa and Middle East	12.68(9.11–16.04)	3.74(2.69–4.73)		28.48(24.24–32.97)	4.57(3.89–5.29)		0.66(0.61–0.7)
High-income North America	11(10.61–11.25)	3.91(3.77–4)		53.05(49.94–55.14)	14.33(13.49–14.9)		4.27(4.15–4.39)

Oceania	0.18(0.11–0.35)	2.81(1.74–5.39)		0.35(0.23–0.63)	2.5(1.64–4.54)		-0.38(-0.47–-0.3)
Central Sub-Saharan Africa	3.03(1.4–5)	5.52(2.55–9.1)		4.05(1.9–8.23)	2.96(1.39–6.01)		-2.01(-2.17–-1.84)
Eastern Sub-Saharan Africa	9.42(5.66–12.44)	4.93(2.97–6.52)		14.46(9.98–20.95)	3.39(2.34–4.92)		-1.21(-1.34–-1.08)
Southern Sub-Saharan Africa	2.09(1.3–3.18)	3.99(2.49–6.07)		5.37(4.51–6.3)	6.69(5.62–7.84)		1.63(1.42–1.83)
Western Sub-Saharan Africa	18.09(10.8–24.63)	9.36(5.59–12.75)		27.85(21.41–34.13)	5.69(4.37–6.97)		-1.61(-1.73–-1.49)
**Mortality**							
High-income Asia Pacific	31.56(28.51–34.49)	18.2(16.44–19.89)		45.31(38.82–50.11)	24.43(20.93–27.02)		0.94(0.69–1.2)
Central Asia	3.76(3.37–4.15)	5.43(4.86–5.99)		5.9(5.01–6.9)	6.16(5.23–7.21)		0.38(0.08–0.67)
East Asia	98.02(82.79–114.92)	8.05(6.8–9.44)		178.49(145.38–218.91)	12.12(9.87–14.86)		1.37(0.89–1.85)
South Asia	13.57(12.26–15.04)	1.24(1.12–1.38)		43.49(39.28–47.98)	2.36(2.13–2.6)		2.08(1.8–2.36)
Southeast Asia	19.34(16.98–22.16)	4.15(3.65–4.76)		43.94(34.6–56.94)	6.29(4.95–8.15)		1.3(1.23–1.38)
Australasia	0.44(0.4–0.47)	2.15(1.98–2.33)		2.59(2.3–2.89)	8.37(7.44–9.33)		4.51(4.06–4.96)
Caribbean	0.55(0.5–0.62)	1.56(1.42–1.75)		1.02(0.88–1.16)	2.14(1.86–2.45)		1.1(0.51–1.69)
Central Europe	4.71(4.35–5.1)	3.76(3.47–4.08)		6.69(6.03–7.44)	5.81(5.23–6.46)		1.43(1.07–1.79)
Eastern Europe	5.02(4.78–5.25)	2.21(2.11–2.32)		8.33(7.73–8.97)	4.03(3.74–4.34)		1.89(1.28–2.5)
Western Europe	20.53(19.44–21.42)	5.34(5.06–5.57)		43.61(39.6–46.26)	9.97(9.05–10.58)		2.00(1.69–2.31)
Andean Latin America	0.58(0.51–0.67)	1.53(1.35–1.77)		1.58(1.22–1.98)	2.4(1.85–3)		1.44(0.72–2.17)
Central Latin America	2.6(2.51–2.68)	1.58(1.52–1.63)		7.56(6.87–8.32)	2.99(2.71–3.29)		1.95(1.72–2.17)
Southern Latin America	0.39(0.35–0.44)	0.79(0.71–0.9)		1.61(1.44–1.76)	2.37(2.13–2.6)		3.69(3.13–4.26)
Tropical Latin America	1.62(1.54–1.67)	1.06(1.01–1.1)		4.89(4.57–5.14)	2.15(2.01–2.26)		2.32(2.1–2.53)
North Africa and Middle East	8.67(6.86–12.02)	2.56(2.02–3.54)		23.68(20.09–27.46)	3.8(3.22–4.41)		1.31(1.18–1.45)
High-income North America	7.01(6.62–7.22)	2.49(2.35–2.57)		28(25.83–29.2)	7.56(6.98–7.89)		3.64(3.33–3.95)

Oceania	0.14(0.09–0.28)	2.16(1.37–4.28)		0.27(0.18–0.49)	1.95(1.28–3.54)		-0.4(-0.54–-0.25)
Central Sub-Saharan Africa	1.85(0.9–3.69)	3.36(1.65–6.71)		3.03(1.47–6.26)	2.21(1.07–4.57)		-1.36(-1.52–-1.21)
Eastern Sub-Saharan Africa	5.13(4.12–6.64)	2.69(2.16–3.48)		9.93(7.53–13.07)	2.33(1.77–3.07)		-0.48(-0.53–-0.43)
Southern Sub-Saharan Africa	1.62(1.02–2.5)	3.09(1.95–4.78)		4.64(3.97–5.44)	5.78(4.94–6.78)		1.99(1.56–2.42)
Western Sub-Saharan Africa	11.87(7.88–17.08)	6.14(4.08–8.84)		19.29(15.82–22.94)	3.94(3.23–4.68)		-1.43(-1.54–-1.31)
**DALYs**							
High-income Asia Pacific	877.09(784.03–967.49)	505.88(452.21–558.02)		839.31(750.07–931.62)	452.59(404.47–502.37)		-0.36(-0.68–-0.05)
Central Asia	114.79(103.02–126.75)	165.62(148.64–182.87)		167.97(142.23–197.86)	175.32(148.45–206.52)		0.17(-0.11–0.45)
East Asia	3400.33(2860.13–3991.71)	279.3(234.93–327.88)		5063.8(4074.24–6285.12)	343.83(276.64–426.75)		0.79(0.39–1.2)
South Asia	469.59(426.81–516.43)	42.95(39.03–47.23)		1274.6(1146.36–1411.9)	69.03(62.08–76.46)		1.57(1.3–1.84)
Southeast Asia	631.45(558.09–719.44)	135.65(119.89–154.55)		1287.28(1007.13–1686.53)	184.34(144.22–241.52)		0.95(0.88–1.03)
Australasia	11.4(10.57–12.31)	56.25(52.14–60.7)		58.67(52.5–65.2)	189.49(169.58–210.59)		4.03(3.59–4.46)
Caribbean	14.77(13.48–16.47)	41.86(38.2–46.66)		25.73(22.11–29.71)	54.21(46.58–62.6)		0.91(0.39–1.44)
Central Europe	122.25(112.65–132.29)	97.73(90.05–105.75)		152.06(136.01–169.48)	131.93(118–147.04)		0.98(0.61–1.35)
Eastern Europe	143.78(137.83–150.04)	63.48(60.85–66.25)		208.18(192.39–225.66)	100.69(93.05–109.14)		1.55(0.62–2.48)
Western Europe	470.61(451.83–489.45)	122.42(117.54–127.32)		881.87(819.77–929.9)	201.62(187.42–212.6)		1.58(1.27–1.9)
Andean Latin America	18.42(16.21–21.14)	48.48(42.67–55.65)		39.83(30.6–50.17)	60.23(46.26–75.86)		0.78(0.29–1.27)
Central Latin America	76.75(74.64–78.92)	46.68(45.4–48)		185.65(167.69–204.99)	73.38(66.28–81.03)		1.31(1.08–1.55)
Southern Latin America	10.13(9.02–11.52)	20.44(18.22–23.25)		37.43(33.71–41.09)	55.29(49.8–60.7)		3.38(2.82–3.93)
Tropical Latin America	49.48(47.59–51.45)	32.43(31.19–33.73)		125.61(119.01–131.67)	55.21(52.3–57.87)		1.78(1.55–2.01)
North Africa and Middle East	272.36(221.56–361.8)	80.3(65.32–106.66)		673.29(567.57–783.68)	108.07(91.1–125.79)		0.99(0.86–1.12)
High-income North America	171.19(164.91–175)	60.83(58.6–62.19)		646.4(610.4–670.7)	174.62(164.89–181.19)		3.46(3.19–3.72)

Oceania	4.79(2.98–9.51)	73.2(45.54–145.21)		8.99(5.88–16.56)	64.56(42.21–118.9)		-0.45(-0.6–-0.31)
Central Sub-Saharan Africa	64.17(32.29–120.45)	116.75(58.74–219.15)		99.41(47.6–209.36)	72.6(34.76–152.9)		-1.53(-1.66–-1.4)
Eastern Sub-Saharan Africa	180.12(145.2–230.08)	94.39(76.09–120.57)		325.43(242.05–438.43)	76.37(56.81–102.89)		-0.69(-0.75–-0.63)
Southern Sub-Saharan Africa	53.47(33.95–82.76)	102(64.76–157.88)		144.31(121.23–170.75)	179.71(150.96–212.63)		1.91(1.6–2.23)
Western Sub-Saharan Africa	396.72(268.1–554.99)	205.39(138.8–287.32)		641.82(515.9–773.75)	131.03(105.32–157.96)		-1.44(-1.56–-1.32)

Note: AAPC: Average annual percentage changes; DALYs: Disability-adjusted life-years; UI: Uncertainty interval.

Table S3. Changes in disability-adjusted life-years (DALYs) number of liver cancer according to population-level determinants of aging, population growth, and epidemiological change from 1990 to 2021 at the global level and by socio-demographic index (SDI) quintile.

**Location**	**Overall difference** [a]	**Change due to Population-level determinants (% contribute to the total changes)**		
		**Epidemiologic changes** [c]	**Population growth** [b]	**Population ageing** [d]
**Global**	5,333,986	-1,608,177 (-30.15%)	3,909,350 (73.29%)	3,032,812 (56.86%)
**Low SDI**	395,207	-225,168 (-56.97%)	614,776 (155.56%)	5,600 (1.42%)
**Low-middle SDI**	991,242	11,418 (1.15%)	637,780 (64.34%)	342,043 (34.51%)
**Middle SDI**	2,026,049	-982,610 (-48.5%)	1,290,720 (63.71%)	1,717,939 (84.79%)
**High-middle SDI**	966,649	-410,569 (-42.47%)	483,521 (50.02%)	893,697 (92.45%)
**High SDI**	952,204	-103,892 (-10.91%)	421,403 (44.26%)	634,693 (66.65%)

a. Change in DALYs number during 1990-2021;

b. Change in DALYs number due to change in population number;

c. Change in DALYs number due to change in epidemiologic changes. Epidemiologic changes refer to the DALYs number change when age structure and population hold constant;

d. Change in DALYs number due to the age structure.

Table S4. Changes in disability-adjusted life-years (DALYs) number of liver cancer due to hepatitis B virus (HBV) according to population-level determinants of aging, population growth, and epidemiological change from 1990 to 2021 at the global level and by socio-demographic index (SDI) quintile.

**Location**	**Overall difference** [a]	**Change due to Population-level determinants (% contribute to the total changes)**		
		**Epidemiologic changes** [c]	**Population growth** [b]	**Population ageing** [d]
**Global**	1,920,020	-1,237,523 (-64.45%)	2,155,205 (112.25%)	1,002,338 (52.2%)
**Low SDI**	166,591	-107,060 (-64.27%)	280,862 (168.59%)	-7,211 (-4.33%)
**Low-middle SDI**	308,237	-41,866 (-13.58%)	273,672 (88.79%)	76,431 (24.8%)
**Middle SDI**	934,415	-765,403 (-81.91%)	942,008 (100.81%)	757,810 (81.1%)
**High-middle SDI**	431,704	-258,057 (-59.78%)	340,378 (78.85%)	349,383 (80.93%)
**High SDI**	78,407	-179,420 (-228.83%)	142,033 (181.15%)	115,793 (147.68%)

a. Change in DALYs number during 1990-2021;

b. Change in DALYs number due to change in population number;

c. Change in DALYs number due to change in epidemiologic changes. Epidemiologic changes refer to the DALYs number change when age structure and population hold constant;

d. Change in DALYs number due to the age structure.

Table S5. Changes in disability-adjusted life-years (DALYs) number of liver cancer due to hepatitis C virus (HCV) according to population-level determinants of aging, population growth, and epidemiological change from 1990 to 2021 at the global level and by socio-demographic index (SDI) quintile.

**Location**	**Overall difference [a]**	**Change due to Population-level determinants (% contribute to the total changes)**		
		**Epidemiologic changes [c]**	**Population growth [b]**	**Population ageing [d]**
**Global**	1,526,665	-242,890 (-15.91%)	1,033,999 (67.73%)	735,556 (48.18%)
**Low SDI**	85,327	-28,746 (-33.69%)	121,103 (141.93%)	-7,030 (-8.24%)
**Low-middle SDI**	333,133	21,567 (6.47%)	222,949 (66.92%)	88,617 (26.6%)
**Middle SDI**	457,596	-83,277 (-18.2%)	247,815 (54.16%)	293,057 (64.04%)
**High-middle SDI**	265,351	-61,832 (-23.3%)	128,294 (48.35%)	198,889 (74.95%)
**High SDI**	384,496	-47,630 (-12.39%)	187,224 (48.69%)	244,901 (63.69%)

a. Change in DALYs number during 1990-2021;

b. Change in DALYs number due to change in population number;

c. Change in DALYs number due to change in epidemiologic changes. Epidemiologic changes refer to the DALYs number change when age structure and population hold constant;

d. Change in DALYs number due to the age structure.

Table S6. Changes in disability-adjusted life-years (DALYs) number of liver cancer due to alcohol use according to population-level determinants of aging, population growth, and epidemiological change from 1990 to 2021 at the global level and by socio-demographic index (SDI) quintile.

**Location**	**Overall difference** [a]	**Change due to Population-level determinants (% contribute to the total changes)**		
		**Epidemiologic changes** [c]	**Population growth** [b]	**Population ageing** [d]
**Global**	1,273,911	75,660 (5.94%)	778,550 (61.11%)	419,701 (32.95%)
**Low SDI**	70,034	-19,462 (-27.79%)	96,287 (137.49%)	-6,792 (-9.7%)
**Low-middle SDI**	206,918	39,993 (19.33%)	131,031 (63.32%)	35,895 (17.35%)
**Middle SDI**	474,410	33,710 (7.11%)	237,303 (50.02%)	203,397 (42.87%)
**High-middle SDI**	193,993	-37,289 (-19.22%)	109,057 (56.22%)	122,226 (63.01%)
**High SDI**	327,696	78,114 (23.84%)	125,752 (38.37%)	123,830 (37.79%)

a. Change in DALYs number during 1990-2021;

b. Change in DALYs number due to change in population number;

c. Change in DALYs number due to change in epidemiologic changes. Epidemiologic changes refer to the DALYs number change when age structure and population hold constant;

d. Change in DALYs number due to the age structure.

Table S7. Changes in disability-adjusted life-years (DALYs) number of liver cancer due to NASH according to population-level determinants of aging, population growth, and epidemiological change from 1990 to 2021 at the global level and by socio-demographic index (SDI) quintile.

Location	Overall difference [a]	Change due to Population-level determinants (% contribute to the total changes)		
		Epidemiologic changes [c]	Population growth [b]	Population ageing [d]
**Global**	591,462	112,143 (18.96%)	319,095 (53.95%)	160,224 (27.09%)
**Low SDI**	49,593	-3,479 (-7.02%)	56,296 (113.52%)	-3,225 (-6.5%)
**Low-middle SDI**	130,923	36,052 (27.54%)	75,974 (58.03%)	18,897 (14.43%)
**Middle SDI**	209,210	10,813 (5.17%)	109,690 (52.43%)	88,707 (42.4%)
**High-middle SDI**	90,153	11,324 (12.56%)	38,348 (42.54%)	40,481 (44.9%)
**High SDI**	111,258	38,535 (34.64%)	35,719 (32.1%)	37,004 (33.26%)

a. Change in DALYs number during 1990-2021;

b. Change in DALYs number due to change in population number;

c. Change in DALYs number due to change in epidemiologic changes. Epidemiologic changes refer to the DALYs number change when age structure and population hold constant;

d. Change in DALYs number due to the age structure.

Table S8. Changes in disability-adjusted life-years (DALYs) number of liver cancer due to hepatoblastoma according to population-level determinants of aging, population growth, and epidemiological change from 1990 to 2021 at the global level and by socio-demographic index (SDI) quintile.

**Location**	**Overall difference [a]**	**Change due to Population-level determinants (% contribute to the total changes)**		
		**Epidemiologic changes [c]**	**Population growth [b]**	**Population ageing [d]**
**Global**	-212,887	-237,603 (-111.61%)	36,201 (17.00%)	-11,485 (-5.39%)
**Low SDI**	-2,193	-60,038 (-2737.14%)	61,719 (2813.78%)	-3,875 (-176.64%)
**Low-middle SDI**	-36,580	-46,263 (-126.47%)	12,655 (34.59%)	-2,972 (-8.12%)
**Middle SDI**	-122,246	-112,581 (-92.09%)	-5,244 (-4.29%)	-4,421 (-3.62%)
**High-middle SDI**	-46,642	-38,763 (-83.11%)	-6,111 (-13.1%)	-1,768 (-3.79%)
**High SDI**	-5,143	-3,949 (-76.8%)	-1,002 (-19.48%)	-192 (-3.73%)

a. Change in DALYs number during 1990-2021;

b. Change in DALYs number due to change in population number;

c. Change in DALYs number due to change in epidemiologic changes. Epidemiologic changes refer to the DALYs number change when age structure and population hold constant;

d. Change in DALYs number due to the age structure.

Table S9. Changes in disability-adjusted life-years (DALYs) number of liver cancer due to other causes according to population-level determinants of aging, population growth, and epidemiological change from 1990 to 2021 at the global level and by socio-demographic index (SDI) quintile.

**Location**	**Overall difference** [a]	**Change due to Population-level determinants (% contribute to the total changes)**		
		**Epidemiologic changes** [c]	**Population growth** [b]	**Population ageing** [d]
**Global**	234,815	-77,587 (-33.04%)	215,816 (91.91%)	96,585 (41.13%)
**Low SDI**	25,856	-6,645 (-25.7%)	33,416 (129.24%)	-914 (-3.54%)
**Low-middle SDI**	48,612	3,966 (8.16%)	36,257 (74.58%)	8,389 (17.26%)
**Middle SDI**	72,665	-63,992 (-88.06%)	77,755 (107%)	58,902 (81.06%)
**High-middle SDI**	32,090	-25,525 (-79.54%)	28,869 (89.96%)	28,747 (89.58%)
**High SDI**	55,490	9,834 (17.72%)	23,692 (42.7%)	21,963 (39.58%)

a. Change in DALYs number during 1990-2021;

b. Change in DALYs number due to change in population number;

c. Change in DALYs number due to change in epidemiologic changes. Epidemiologic changes refer to the DALYs number change when age structure and population hold constant;

d. Change in DALYs number due to the age structure.

Table S10. Changes in disability-adjusted life-years (DALYs) number of liver cancer caused by specific etiologies from 1990 to 2021 at the global level and by socio-demographic index (SDI) quintile

**Location**	**Overall difference** [a]	**Change due to Causes (% contribute to the total changes)**					
		HBV [b]	HCV [c]	**Alcohol use** [d]	**NASH** [e]	**Hepatoblastoma** [f]	**Other causes** [g]
**Global**	5,333,986	1,920,020 (36.00%)	1,526,665 (28.62%)	1,273,911 (23.88%)	591,462 (11.09%)	-212,887 (-3.99%)	234,815 (4.4%)
**Low SDI**	395,207	166,591 (42.15%)	85,327 (21.59%)	70,034 (17.72%)	49,593 (12.55%)	-2,193 (-0.56%)	25,856 (6.54%)
**Low-middle SDI**	991,242	308,237 (31.1%)	333,133 (33.61%)	206,918 (20.87%)	130,923 (13.21%)	-36,580 (-3.69%)	48,612 (4.9%)
**Middle SDI**	2,026,049	934,415 (46.12%)	457,596 (22.59%)	474,410 (23.42%)	209,210 (10.33%)	-122,246 (-6.03%)	72,665 (3.59%)
**High-middle SDI**	966,649	431,704 (44.66%)	265,351 (27.45%)	193,993 (20.07%)	90,153 (9.33%)	-46,642 (-4.83%)	32,090 (3.32%)
**High SDI**	952,204	78,407 (8.23%)	384,496 (40.38%)	327,696 (34.41%)	111,258 (11.68%)	-5,143 (-0.54%)	55,490 (5.83%)

Note: HBV: Hepatitis B virus; HCV: Hepatitis C virus; NASH: Non-alcohol related steatohepatitis.

a. Change in DALYs number during 1990-2021; b. Change in DALYs number due to change in liver cancer attributed to HBV;

c. Change in DALYs number due to change in liver cancer attributed to HCV;

d. Change in DALYs number due to change in liver cancer attributed to alcohol use;

e. Change in DALYs number due to change in liver cancer attributed to NASH;

f. Change in DALYs number due to change in liver cancer attributed to Hepatoblastoma;

g. Change in DALYs number due to change in liver cancer attributed to other causes.
